# Modification of the *Streptococcus mutans* transcriptome by LrgAB and environmental stressors

**DOI:** 10.1099/mgen.0.000104

**Published:** 2017-02-28

**Authors:** Kelly C. Rice, Matthew E. Turner, O’neshia V. Carney, Tongjun Gu, Sang-Joon Ahn

**Affiliations:** ^1^​ Department of Microbiology and Cell Science, Institute of Food and Agricultural Sciences, University of Florida, Gainesville, FL 32611, USA; ^2^​ Bioinformatics, Interdisciplinary Center for Biotechnology Research, University of Florida, Gainesville, FL 32610, USA; ^3^​ Department of Oral Biology, College of Dentistry, University of Florida, Gainesville, FL 32610, USA; ^†^​ Present address: Department of Health Outcomes and Policy, College of Medicine, University of Florida, Gainesville, FL 32610, USA.

**Keywords:** *S. mutans*, oxygen, heat-shock, vancomycin, stress response, LrgAB

## Abstract

The *Streptococcus mutans* Cid/Lrg system is central to the physiology of this cariogenic organism, affecting oxidative stress resistance, biofilm formation and competence. Previous transcriptome analyses of *lytS* (responsible for the regulation of *lrgAB* expression) and *cidB* mutants have revealed pleiotropic effects on carbohydrate metabolism and stress resistance genes. In this study, it was found that an *lrgAB* mutant, previously shown to have diminished aerobic and oxidative stress growth, was also much more growth impaired in the presence of heat and vancomycin stresses, relative to wild-type, *lrgA* and *lrgB* mutants. To obtain a more holistic picture of LrgAB and its involvement in stress resistance, RNA sequencing and bioinformatics analyses were used to assess the transcriptional response of wild-type and isogenic *lrgAB* mutants under anaerobic (control) and stress-inducing culture conditions (aerobic, heat and vancomycin). Hierarchical clustering and principal components analyses of all differentially expressed genes revealed that the most distinct gene expression profiles between *S. mutans* UA159 and *lrgAB* mutant occurred during aerobic and high-temperature growth. Similar to previous studies of a *cidB* mutant, *lrgAB* stress transcriptomes were characterized by a variety of gene expression changes related to genomic islands, CRISPR-C as systems, ABC transporters, competence, bacteriocins, glucosyltransferases, protein translation, tricarboxylic acid cycle, carbohydrate metabolism/storage and transport. Notably, expression of *lrgAB* was upregulated in the wild-type strain under all three stress conditions. Collectively, these results demonstrate that mutation of *lrgAB* alters the transcriptional response to stress, and further support the idea that the Cid/Lrg system acts to promote cell homeostasis in the face of environmental stress.

## Abbreviations

AB, lrgAB mutant strain; Cas, CRISPR-associated; CRISPR, clustered regularly interspaced short palindromic repeats; DE, differential expression; GI, genomic island; GO, gene ontology; PCA, principal component analysis; PTS, phosphotransferase system; qRT-PCR, quantitative real-time PCR; RNA-seq, RNA sequencing; RPKM, reads per kilobase of transcript per million mapped reads; T/A, toxin/antitoxin; TCA, tricarboxylic acid.

## Data Summary

All raw RNA-seq data analysed in this study have been deposited in the National Center for Biotechnology Information (NCBI) Gene Expression Omnibus (www.ncbi.nlm.nih.gov/geo/) and are accessible through GEO Series accession number GSE84427 (www.ncbi.nlm.nih.gov/geo/query/acc.cgi?token=apyjiygsvzarnsv&acc=GSE84427).

## Impact Statement

Cid and Lrg membrane proteins are ubiquitous in bacteria. Although their contribution to cell death, antibiotic tolerance, biofilm formation and/or competence has been well documented in two model Gram-positive organisms, *Streptococcus* *mutans* and *Staphylococcus aureus*, clues to their specific cellular functionality are relatively scarce. Comparison of wild-type and *lrgAB* mutant transcriptomes under several stress conditions therefore provides a valuable opportunity to detect gene expression changes unique to the *lrgAB* mutant. This is an important step in determining if one or more of these changes directly or indirectly contributes to the *lrgAB* mutant growth impairment observed under each stress, and whether there is an *S. mutans* core transcriptome response to all three stresses. Additionally, assessing stress-specific gene expression patterns common to both *lrgAB* mutant and wild-type under each growth condition reveals previously undefined *S. mutans* heat and vancomycin stress transcriptomes. Combined with our recently published transcriptome profiling of an *S. mutans cidB* mutant, the studies documented herein provide further support for our evolving hypothesis that the bacterial Cid and Lrg membrane proteins (previously suggested to be involved in regulation of cell death) may act as redundant systems that contribute to or regulate cell homeostasis in response to exogenous stress in *S. mutans*.

## Introduction

*Streptococcus mutans*, the primary agent of human dental caries [1], employs a variety of survival strategies to establish itself within the oral cavity. These include competition for nutrients and physical real estate within dental plaque biofilm, as well as adaptation to fluxes in environmental pH, temperature and oxidative stress (reviewed in [2, 3]). The *S. mutans lrgA/B* and *cidA/B* genes have been previously demonstrated to affect the growth and stress response of *S. mutans in vitro* [4–6]. These operons are ubiquitous among a variety of Gram-positive and Gram-negative bacteria, with the ‘A’ genes predicted to encode membrane proteins bearing predicted structural similarity to bacteriophage lambda holins (reviewed in [7–9]). These shared features include a relatively small size, two to three membrane-spanning domains, and highly charged N and C termini [10, 11]. During bacteriophage lytic infection, holins control the timing and onset of host cell lysis. In *Staphylococcus aureus*, LrgAB was proposed to inhibit the function of CidAB (holin) in a manner analogous to that of a bacteriophage antiholin. However, it is still unclear whether CidA and LrgA proteins in both *S. mutans* and *S.* *aureus* function in a similar manner. This being said, a body of mutational analysis of the Cid/Lrg system in *S.* *aureus* supports the involvement of these operons in carbohydrate metabolism, antibiotic tolerance, biofilm formation and cell death/lysis [10–16]. The CidB and LrgB proteins are also predicted to be integral membrane proteins, but they do not contain other domains of known function, and do not appear to be structurally similar to holins [10, 11]. Genes homologous to *S.* *aureus cidAB* and *lrgAB* have been identified in a variety of bacteria and archaea [8], but have only been characterized in a few other bacteria such as *S. mutans* [4–6] and *Bacillus anthracis* [17, 18].

Our previous studies have also defined an important role for the *S. mutans cid* and *lrg* operons in this organism’s physiology, stress tolerance and biofilm-forming ability [4–6]. Similar to previous reports of *S.* *aureus cid* regulation [11, 19–21], expression of both *S. mutans cidAB* and *lrgAB* are differentially regulated in response to culture aeration [4, 22] and glucose levels [4, 6]. The LytSR/LytST two-component regulator has also been shown to positively regulate *lrgAB* expression in both organisms [4, 6, 23]. Furthermore, *S. mutans cidB*, *cidAB* and *lrgAB* mutants display a pronounced sensitivity to oxidative stress, as has been demonstrated by aerated growth in both agitated liquid cultures [5] and on agar plates [6], as well as during growth in the presence of paraquat [6] and hydrogen peroxide [4]. Various *S. mutans lrg* mutants have also displayed alterations in competence [4] and biofilm formation [6]. Of particular note, an *S. mutans lrgA* mutant had a significantly impaired ability to be transformed by exogenous plasmid DNA [4] and a reduced capacity to form biofilms [6].

Recently, RNA sequencing (RNA-seq) comparison of *S. mutans* UA159 and its isogenic *cidB* mutant under both aerobic and anaerobic growth conditions has been performed [5]. In addition to predicted changes in oxidative-stress-related responses, this analysis revealed a potential link between CidB and expression of a number of extrachromosomal element genes, including those belonging to genomic islands (GIs) TnSMu1, TnSMu2 and CRISPR (clustered regularly interspaced short palindromic repeats)-CRISPR-associated (Cas) systems [5]. It has been shown by others that genes from both TnSMu2 and CRISPR1-Cas contribute to resistance to oxidative stress, whereas CRISPR2-Cas appears to be involved in the *S. mutans* response to heat shock [24]. Notably, altered expression of all three of these GIs has also been associated with perturbation of *S. mutans* ClpP, an intracellular proteolytic complex which modulates stress response and misfolded protein turnover [25]. This apparent parallel in the transcriptional responses of *S. mutans* to *clpP* and *cidB* mutation suggests that the Cid/Lrg system, like ClpP, may represent an important modulator of cell homeostasis and/or general stress resistance. To further address this hypothesis, we have used RNA-seq and bioinformatics analyses in the current study to assess the transcriptional response of the wild-type and isogenic *lrgAB* mutant to growth in the presence of three different stress conditions (aerobiosis, high temperature and the cell-wall active antibiotic vancomycin). It was anticipated that this comprehensive transcriptomic study would help to clarify the *S. mutans* cellular response and role of the *lrgAB* operon in responding to environmental stressors encountered in the oral cavity.

## Methods

### Bacterial strains

The bacterial strains used in this study were *S. mutans* UA159, a serotype c strain [26], and previously published isogenic *lrgA*, *lrgB* and *lrgAB* mutants [6], which were all created using the PCR ligation mutagenesis technique [27]. All *S. mutans* stock cultures were maintained at −80 °C in 50 % (v/v) glycerol. For each experiment, UA159 and each mutant was streaked on brain heart infusion (BHI) agar plates, containing either 1000 µg spectinomycin ml^−1^ (*lrgA* mutant), 10 µg erythromycin ml^−1^ (*lrgB* mutant) or 1000 µg kanamycin ml^−1^ (*lrgAB* mutant). Agar plates were grown for 48 h at 37 °C and 5 % CO_2_ prior to sub-culturing of individual colonies in BHI broth and overnight growth at 37 °C and 5 % CO_2_.

### Growth curve stress experiments

To compare growth of wild-type UA159 and its isogenic *lrgA*, *lrgB* and *lrgAB* mutants under conditions of heat stress and vancomycin supplementation, overnight cultures of each strain were inoculated into sterile BHI broth at a 1 : 100 dilution, and the optical density at 600 nm (OD_600_) was monitored using a Bioscreen C lab system as detailed elsewhere [28]. Heat stress and vancomycin growth curves were performed using anaerobic conditions, whereby sterile mineral oil (50 µl per well) was additionally placed on top of the cultures. For growth under heat stress, temperature was raised to 42 °C. For growth under vancomycin treatment, BHI medium was supplemented with vancomycin (Sigma) at a final concentration of 1 µg ml^−1^. Oxidative stress growth assays were performed by supplementing each culture with 1 mM H_2_O_2_ and growing in a Biotek Synergy HT microplate reader, as described previously [4].

### Culture growth for RNA isolation

Wild-type strain UA159 and Δ*lrgAB* overnight cultures were each diluted 1 : 50 in sterile BHI broth and grown to mid-exponential growth phase (OD_600_ of 0.4) under four different environmental conditions (anaerobic, aerobic, heat stress, vancomycin stress), as follows. For anaerobic growth, including heat and vancomycin stress conditions, sterile mineral oil was placed on top of the cultures. For aerobic growth, each culture was grown in a 250 ml conical flask at a 1 : 5 volume to flask ratio, and incubated at 115 r.p.m. and 37 °C. For heat stress growth, cultures were incubated at 40 °C [see Fig. S1 (available in the online Supplementary Material) for corresponding growth curve]. For vancomycin growth, cultures were supplemented with 1 µg vancomycin ml^−1^ and incubated at 37 °C. Growth curves of the wild-type and *lrg* mutants corresponding to the aerobic culture condition have been previously published [5].

### RNA-seq and differential expression (DE) analysis

Total RNA was isolated as described previously [4, 5, 22] from wild-type strain UA159 and *lrgAB* mutant cell cultures grown to an OD_600_ of 0.4 as described above. For each growth condition (anaerobic, aerobic, heat stress, vancomycin stress), total RNA was isolated from *n*=3 independent cultures each for the wild-type and *lrgAB* mutant. RNA processing (including 16S/23S removal and cDNA library creation) was performed on 5 µg of high-quality total RNA (*A*_260/280_ >2.0) as described previously [5]. Among the obtained 1 µg enriched mRNAs, 100 ng was subjected to cDNA library preparation using the NEBNext Ultra Directional RNA library prep kit (New England Biolabs). The final concentration and quality of each cDNA library was analysed on an Agilent TapeStation (Agilent Technologies) prior to Illumina NextSeq500 sequencing (single-read format) by the NextGen DNA Sequencing Core Laboratory, ICBR, at the University of Florida (Gainesville, FL, USA). Read mapping was performed on a Galaxy server hosted by the research computing centre at the University of Florida, using Bowtie for Illumina (version 1.1.2). Mapped reads per gene were then counted from BAM files using htseq-count, as described previously [29]. Count-based differential expression analysis of RNA-seq data was performed with the R package edgeR on RStudio, as described elsewhere [30]. Briefly, a table of read counts of all the ORFs, excluding all rRNA and tRNA genes, was uploaded into RStudio and then the statistical procedure of edgeR (available as packages of the Bioconductor software development project [31]) was used to call DE genes. The fold-change expression in a subset of DE genes was also assessed in all RNA samples by quantitative real-time PCR (qRT-PCR) using the primers listed in Table S1, using previously described methodologies [4, 5, 22]. All raw RNA-seq data have been deposited in NCBI’s Gene Expression Omnibus (GEO) [32] and are accessible through GEO Series accession number GSE84427.

### Hierarchical clustering, principal component analysis (PCA) and generation of Venn diagrams

The programming language R was used for all statistical analyses. For both hierarchical clustering and PCA, the reads per kilobase of transcript per million mapped reads (RPKM) were calculated for all genes identified in all 10 pairwise DE analyses that met the following criteria: the gene was annotated with gene length, ≥2.0-fold change, false discovery rate *P*≤0.005 and the gene had a read count of 72 or more across all the samples in each comparison. Both hierarchical clustering and PCA are unsupervised learning methods that can be used to examine the broad pattern of DE genes to uncover the features of all the samples within conditions as well as across conditions. For hierarchical clustering analysis, the Euclidean distance between samples was calculated and used for measuring the similarity between samples. The agglomerative clustering method was then used for clustering the samples based on the average distance between the sample and existing cluster, and the resulting heatmap and dendrogram were visualized using gplots. Similar to clustering, PCA also partitions the samples into different groups, but uses a low-dimensional space to represent the data. For this study, the principal components were extracted by orthogonal transformation and represent the highest variance in the data, thus filtering potential noise and allowing a clearer pattern to be observed. For generation of Venn diagrams, the number of DE genes overlapping between each comparison was calculated using a fold-change cutoff of either 2.0- or 1.5-fold, as indicated in the Results. Venn diagrams were then visualized using the VennDiagram package [33] in R.

### Functional analysis of DE data

Gene ontology (GO) enrichment was performed with the DAVID (version 6.7) Functional Annotation Clustering Tool [34, 35] using default analysis parameters. Functional clusters highlighted in this study were selected if their enrichment score (value representative of the total geometric mean of all terms included within the enriched group followed by a negative log transformation) was greater than 1.3, which corresponds to a non-log *P*-value of 0.05 [34]. The DAVID analysis was performed on DE genes using the following cutoffs: *P*<0.005 and a fold-change cutoff of either 2.0- or 1.5-fold, as indicated in the Results. Where applicable, REVIGO [36] was also used to reduce redundancy of GO terms in each DAVID functional cluster. Gene annotation/functional categories presented in all Tables and DE Supplemental Files were obtained using PATRIC [37], UniProt [38] and/or the NCBI *S. mutans* UA159 reference genome (NC_004350.2).

## Results and discussion

### Response of the *lrgAB* mutant to environmental stressors

**Fig. 1.**
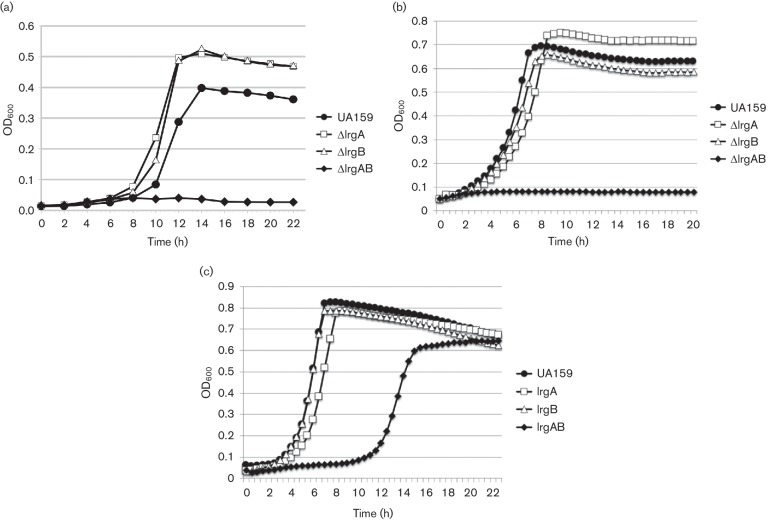
Inhibitory effect of *lrgAB* mutation on *S. mutans* growth in the presence of 1 mM H_2_O_2_ (a), at 42 °C (b) or in the presence of 1 µg vancomycin ml^−1^ (c). Growth curves were obtained by growing the wild-type and each indicated mutant in a Biotek plate reader (a) or in a Bioscreen C (b and c), as described in Materials and Methods. Data are representative of *n*=3 independent experiments.

Previously, our research demonstrated that mutation of *lrgAB* in *S. mutans* rendered this bacterium more sensitive to oxidative stress, whereas *lrgA* and *lrgB* mutants grew similarly to the wild-type strain under this same condition [5, 6]. To verify this phenotype, wild-type and isogenic *lrg* mutants were grown in parallel in the presence of 1 mM H_2_O_2_ (Fig. 1a). As previously observed [4], the *lrgAB* mutant did not display appreciable growth under this culture condition (Fig. 1a). Interestingly, the *lrgA* and *lrgB* mutants each displayed modestly enhanced growth relative to the wild-type in the presence of H_2_O_2_ (Fig. 1a). To determine if impaired growth of the *lrgAB* mutant was specific to oxidative stress, the ability of these mutants to grow anaerobically at a high suboptimal growth temperature (42 °C; Fig. 1b) was also assessed. In this assay, the *lrgAB* mutant displayed a greater sensitivity to heat stress relative to the wild-type, *lrgA* and *lrgB* mutant strains. Since the *cid* and *lrg* operons contribute to antibiotic tolerance in *S. aureus* [10, 11], the *lrgA*, *lrgB* and *lrgAB* mutants were also tested for growth in the presence of vancomycin, a cell-wall active glycopeptide (Fig. 1c). Similar to growth trends during aerobic and high-temperature growth, the *lrgAB* mutant displayed impaired growth in the presence of vancomycin, whereas the *lrgA* and *lrgB* mutant growth curves were similar to that of the wild-type (Fig. 1c). Growth of all *lrg* mutants was previously shown to be as robust as that of the wild-type strain under 37 °C non-stressed anaerobic growth [6]. Thus, it seems to be clear that unlike the *cidAB* operon [5, 6], both ‘A’ and ‘B’ components are required for the ability of *S. mutans* to cope with those stressors in the *lrg* operon. However, we failed to complement the loss of *lrgAB* by simply producing LrgAB *in trans.* Furthermore, considering our previous observation that the function of the ‘B’ component (CidB) could be functionally checked by the ‘A’ components (CidA and LrgA) [5], it is possible that the mutation of *lrgAB* may be linked to the function of CidAB as a redundant system in *S. mutans.* Nevertheless, the fact that *lrgAB* mutation confers increased sensitivity to these three different stress conditions implies that this operon may play a common or overlapping regulatory role in the cell’s general response to stress. Alternatively, these results may suggest that mutation of *lrgAB* impacts a critical aspect of physiology, the disruption of which makes the cell more sensitive to growth under suboptimal/stress conditions.

### RNA-seq analyses of wild-type and *lrgAB* mutant transcriptomes

To better understand how mutation of *lrgAB* impacts the ability of *S. mutans* to grow under stress conditions, RNA extracted from mid-exponential phase anaerobic, aerobic (a), heat stress (h) or vancomycin (v) cultures (*n*=3 biological replicates per strain per growth condition) of UA159 (designated ‘WT’ in all subsequent figures and tables) and isogenic *lrgAB* mutant (designated ‘AB’ in all subsequent figures and tables) was subjected to RNA-seq profiling, as described in Materials and Methods. Because the *lrgAB* mutant was not able to grow in the presence of H_2_O_2_ (Fig. 1a), aerobic growth conditions were used to assess gene expression changes in response to oxidative stress, as this has been previously shown to permit growth (albeit impaired) of the *lrgAB* mutant [5]. Likewise, a 40 °C growth temperature was chosen for heat stress transcriptome profiling, since this temperature permits impaired growth of the *lrgAB* mutant (Fig. S1) as opposed to near-complete growth inhibition (Fig. 1b). In total, 10 DE analyses were performed: four comparing gene expression of the *lrgAB* mutant relative to wild-type under each growth condition, three comparing gene expression of wild-type stress conditions (aerobic, high temperature or vancomycin) relative to wild-type anaerobic growth and three comparing gene expression of *lrgAB* mutant stress conditions relative to *lrgAB* mutant anaerobic growth. To validate the RNA-seq DE results, expression of a subset of 12 genes was also assessed under all growth conditions and pairwise comparisons using qRT-PCR. Overall, qRT-PCR analysis revealed a high degree of conservation between expression patterns of these 12 genes compared to RNA-seq DE analyses (Table S2). Genes from all 10 DE analyses that met the criteria of either ≥1.5- or ≥2.0-fold change in expression (as indicated in each section/analysis below) and *P*≤0.005 were selected for subsequent bioinformatics analyses.

**Fig. 2.**
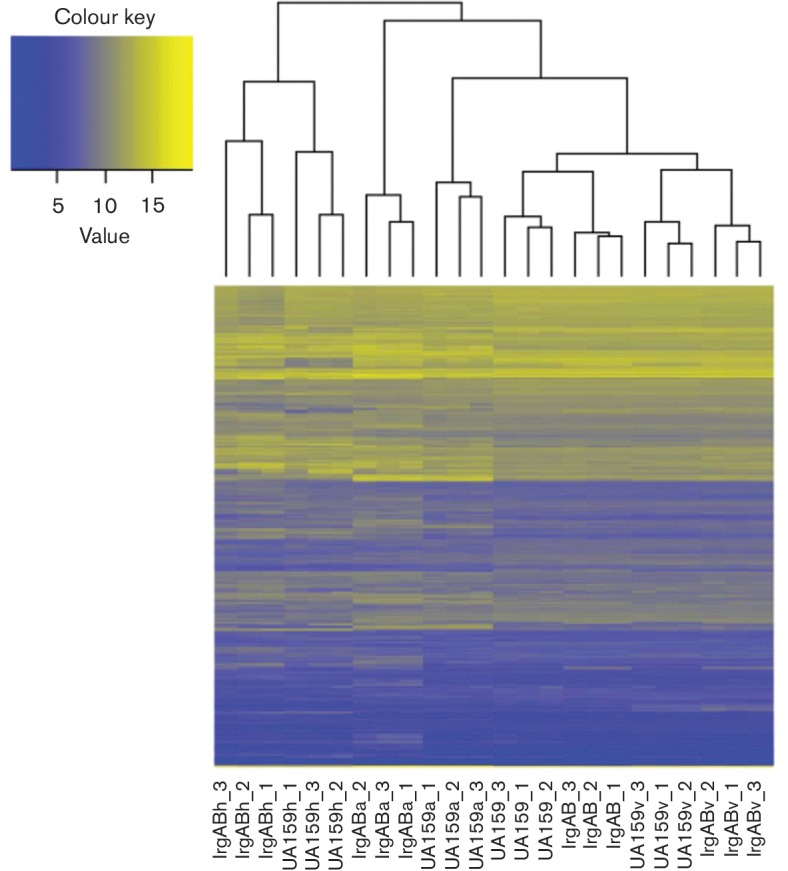
Cluster analysis of RNA-seq data. DE genes with >2.0-fold change and *P*<0.005 were clustered across all the samples based on the similarity calculated by Eucledian distance (indicated by tree at the top of the heat map). The heat map key represents the log2-transformed RPKM. The *y*-axis is the DE genes (unlabelled). Sample names are indicated at the bottom of the heat map (*x*-axis).

To obtain an initial birds-eye view of the RNA-seq DE data, hierarchical clustering analysis of all genes (≥2.0-fold change in expression and *P*≤0.005) identified in the 10 DE analyses listed above was performed (Figs 2 and S2). As expected, the *n*=3 biological replicates for the wild-type and *lrgAB* mutant under each growth condition displayed good clustering within each experimental group. This analysis also suggested that the overall expression trends in the wild-type and *lrgAB* samples obtained from the anaerobic and vancomycin growth conditions were more similar to each other, while the greatest overall separation in DE patterns occurred between the aerobic wild-type and aerobic *lrgAB* mutant samples. Similar to clustering analysis, PCA of all 24 RNA-seq samples (Fig. S3) demonstrated tight clustering within each biological triplicate, as well as general clustering between the wild-type and *lrgAB* mutant under each of the four different growth conditions. This analysis also revealed that the heat stress and aerobic gene expression patterns for both the wild-type and *lrgAB* mutant clustered separately from the anaerobic and vancomycin transcriptomes (Fig. 2). The heat map of all DE genes (≥2.0-fold change in expression and *P*≤0.005) was also performed using the mean RPKM values of the three replicates per strain per growth condition (Fig. S4). This analysis demonstrated a similar pattern as shown in Fig. 2 that the observed DE changes occurred in all low-, middle- and high-expression genes.

### *lrgAB* mutant has a very similar transcriptomic profile as the *cidB* mutant but a different one from the *lytS* mutant during unstressed anaerobic growth

When we compared the transcriptomes of the wild-type and *lrgAB* mutant in the non-stress condition (at 37 °C, anaerobic growth), loss of LrgAB significantly affected the expression of only 22 genes using a 2.0-fold expression change cutoff, and 53 genes (32 downregulated and 21 upregulated; Table S3) using a 1.5-fold expression change cutoff. The majority of these 53 DE genes belonged to very few distinct functional groups, including the GIs TnSmu1 and TnSmu2, the CRISPR-Cas system, bacteriocin production, energy metabolism and amino acid ABC transporters. Notably, these expression profiles were almost identical to those observed previously in an RNA-seq study of wild-type versus *cidB* strain in the same condition [5]. Additionally, SMU_1363c was the most highly upregulated gene in the *lrgAB* strain (about 38-fold; Table S3), as was observed in the *cidB* mutant strain under this growth condition (about 25-fold). Therefore, the stress sensitivity conferred by lack of either CidB or LrgAB may occur through similar mechanisms or pathways. These results also suggest that Cid and Lrg may be functionally linked, or share their downstream pathways, which is consistent with our previous observations [5]. It is also noteworthy that the *lrgAB* mutant showed a quite different transcriptomic profile from the *lytS* mutant [4], given that *lrgAB* is under the control of LytST [6]. In particular, the TnSmu2 genes, including SMU_1354c, SMU_1360c, SMU_1363c and SMU_1365c, were regulated in a completely opposite manner between *lrgAB* (up) and *lytS* (down) strains [4], suggesting that the transcriptomic changes by mutation of *lrgAB* may be independent of LytST. Thus, it is possible that the function of LrgAB could be determined at the post-transcriptional level, and more intimately associated with CidB.

### Gene expression patterns common to wild-type and *lrgAB* mutant in response to aerobic growth

**Fig. 3.**
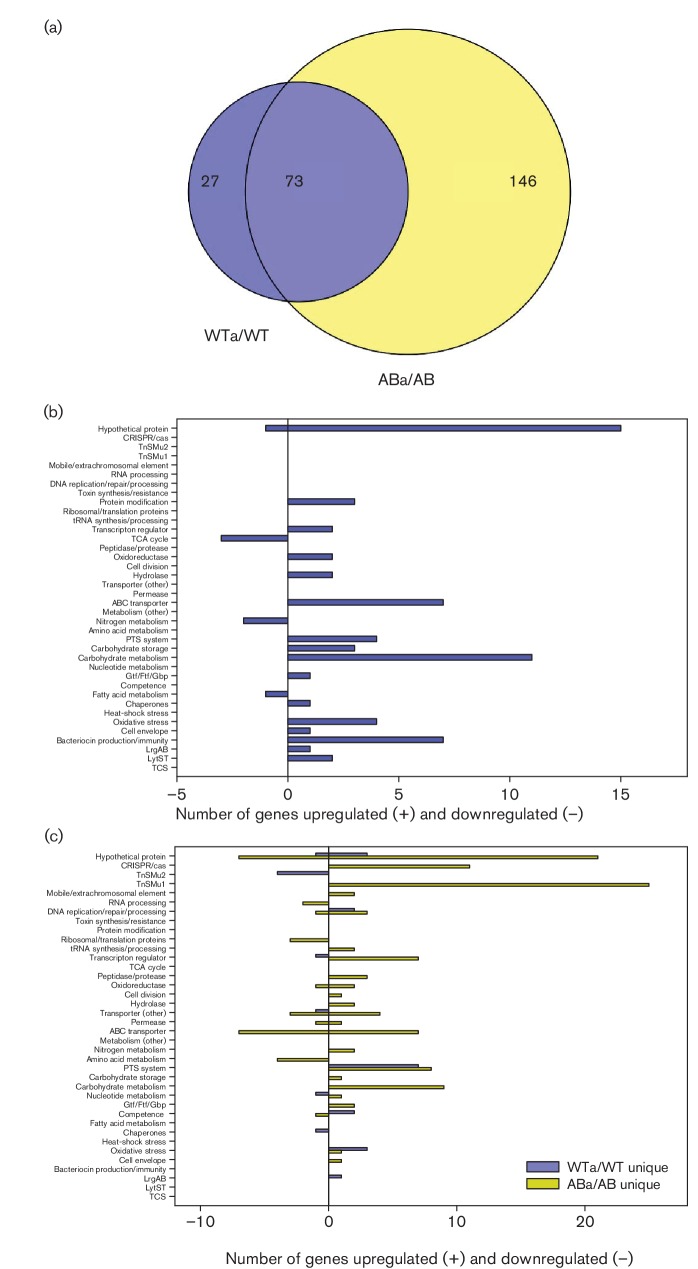
Comparison of wild-type and *lrgAB* mutant gene expression during aerobic growth. (a) Venn diagrams were used to identify overlapping and unique genes in WTa/WT and ABa/AB DE analyses (≥2.0-fold change and *P*<0.005). (b) Functional categorization of 73 overlapping genes between WTa/WT and ABa/AB DE analyses (≥2.0-fold change and *P*<0.005). (c) Functional categorization of 27 and 146 genes unique to WTa/WT (blue bars) and ABa/AB (yellow bars) DE analyses, respectively (≥2.0-fold change and *P*<0.005). For (b) and (c), functional categories were assigned using genomic functional annotations as well as those found in the DAVID and PATRIC databases. Upregulated genes in each category are found to the right of the *y*-axis (positive numbers), and downregulated genes are found to the left of the *y*-axis (negative numbers).

To distinguish between the *S. mutans* general transcriptome response to aerobic growth and gene expression changes that are specific to each of the wild-type and *lrgAB* mutant during aerobic growth, Venn diagrams were used to compare the DE results of *lrgAB* aerobic growth versus *lrgAB* anaerobic growth (ABa/AB) and wild-type aerobic growth versus wild-type anaerobic growth (WTa/WT) (Fig. 3a). Given the large number of gene expression changes observed during aerobic growth, these analyses were performed using a ≥2.0-fold expression change cutoff. This analysis revealed that 73 genes were common to both WTa/WT and ABa/AB DE comparisons, a subset which probably represents the general gene expression changes in response to aerobic stress. The bulk of these genes (*n*=65) were upregulated relative to anaerobic growth in both the wild-type and *lrgAB* mutant cultures, and fell primarily into the categories of hypothetical proteins, transcriptional regulators, carbohydrate metabolism/storage/transport proteins, ABC transporters and biofilm-related genes (glucosyltransferases, glucan-binding proteins) (Fig. 3b and Table S4). Interestingly, upregulated aerobic expression of the *lrgA* transcript was detected in both the wild-type and *lrgAB* mutant strains (Table S4). However, the bulk of the *lrgA* reads in the *lrgAB* mutant dataset aligned to the 5′ region of the gene that was not replaced by the kanamycin resistance gene, when visualized in Integrated Genome Viewer. Of the eight common downregulated genes in response to aerobic stress, three encoded tricarboxylic acid (TCA) cycle enzymes (aconitase, citrate synthase, isocitrate dehydrogenase), two were involved in ammonium transport and three encoded hypothetical proteins (Table S4). The three TCA cycle enzymes are encoded as an operon (SMU_670–SMU_672) and also represent the first three steps of glutamate/glutamine biosynthesis. Interestingly, expression of these genes has been previously reported to be downregulated in response to acid stress, an adaptation which was proposed to minimize synthesis of amino acid precursors and promote citrate metabolism to pyruvate, which in turn would allow H^+^ consumption and ATP synthesis [39]. It is therefore possible that a similar adaptation helps *S. mutans* deal with aerobic stress.

DAVID and REVIGO functional annotation clustering analysis of these 73 overlapping genes revealed five significant GO term enrichments, including TCA cycle/pyruvate metabolism, glycolysis/gluconeogenesis, oxidoreductases, cellular homeostasis, electron carrier activity and starch/sucrose metabolism (Table 1). Additionally, several of the most highly expressed genes in both the wild-type and *lrgAB* mutant in response to aerobic growth were operons encoding bacteriocin-related proteins (Fig. 3b and Table S4). These included SMU_423 (*nlmD*), SMU_150–SMU_153 (non-lantibiotic mutacin IV operon) and SMU_1903c–SMU_1914c (encoding a variety of hypothetical proteins, predicted bacteriocins, immunityproteins and secretion proteins). Genes previously implicated in the response or resistance of *S. mutans* to oxidative stress were also identified in this subset of 73 genes, including those encoding ClpB ATP-dependent protease [22], alkyl hydroperoxide reductase [22], H_2_O-forming NADH oxidase [22], superoxide dismutase [22] and LytST [4]. Overall, these gene expression changes in response to aerobic growth common to both the wild-type and *lrgAB* mutant were very similar to those observed previously in an RNA microarray study comparing *S. mutans* gene expression during aerobic and anaerobic growth [22].

**Table 1.** Summary of DAVID functional annotation clustering analysis of unique and common genes under aerobic, heat stress and vancomycin growth (corresponding to data presented in Figs 3, 4 and 6) Enrichment scores of ≥1.33 (corresponding to *P*<0.05) are reported. PTS, phosphotransferase system.

Growth condition	Venn comparison	Enrichment score	GO keywords/categories
Aerobic growth (2.0-fold expression change cutoff)	WTa/WT & ABa/AB overlap	4.33	Pyruvate metabolism; glycolysis/gluconeogenesis
		3.15	Butanoate metabolism; oxidoreductases
		2.23	Glycolysis/gluconeogenesis; lipoic acid binding; carboxylic acid binding
		1.87	Cellular homeostasis; electron carrier activity; pyridine nucleotide-disulphide oxidoreducase
		1.69	Starch and sucrose metabolism; cellular carbohydrate biosynthetic process
	WTa/WT unique	2.97	Secondary metabolites biosynthesis, transport and catabolism; acyl carrier activity; amino acid adenylation
		1.44	Carbohydrate transport; PTS
	ABa/AB unique	1.65	Carbohydrate transport; PTS; fructose and mannose metabolism
Heat stress growth (2.0-fold expression change cutoff)	WTh/WT & ABh/AB overlap	2.83	Secondary metabolites biosynthesis, transport and catabolism; acyl carrier activity; amino acid adenylation
		1.50	Starch and sucrose metabolism; cellular carbohydrate biosynthetic process
	WTh/WT unique	N/A	No functional clusters identified
	ABh/AB unique	6.56	Ribosome; rRNA binding, RNA binding; translation
Vancomycin growth (1.5-fold expression change cutoff)	WTv/WT & ABv/AB overlap	1.60	Pyruvate metabolism; glycolysis/gluconeogenesis
	WTv/WT unique	2.31	TCA cycle; cofactor catabolic process
	ABv/AB	2.11	Lactose/galactose metabolism
	unique	1.79	Starch and sucrose metabolism; cellular carbohydrate biosynthetic process
Wild-type overlapped genes (all three conditions; 1.5-fold expression change cutoff)	WTa/WT,	3.27	TCA cycle; pyruvate metabolism; glycolysis
	WTh/WT, & WTv/WT overlap	2.81	TCA cycle; cofactor catabolic process
*lrgAB* mutant overlapped genes (all three conditions; 1.5-fold expression change cutoff)	ABa/AB	2.13	Lactose metabolism
	ABh/AB	2.08	Cell envelope
	ABv, AB overlap	1.91	Starch and sucrose metabolism; cellular carbohydrate biosynthetic process
		1.76	TCA cycle; pyruvate metabolism; glycolysis
		1.52	Oxidation reduction; energy production and conversion

### Gene expression patterns unique to wild-type and *lrgAB* mutant in response to aerobic growth

To gain insight into the growth defect of the *lrgAB* mutant in response to oxidative stress, the unique (non-overlapping) gene expression changes in response to aerobic growth (≥2.0-fold expression change cutoff) were compared between the wild-type and *lrgAB* mutant (Fig. 3a, c). Interestingly, there were many more unique gene expression changes observed in the *lrgAB* mutant (115 upregulated, 31 downregulated) compared to the wild-type strain (19 upregulated, eight downregulated) (Tables S5 and S6). Notable gene expression changes unique to the wild-type strain that have been previously implicated in oxidative stress resistance include downregulated expression of SMU_1339–SMU_1342 [40], and upregulated expression of *comDE* [22], *tpx* [41], *gshR* [42], SMU_1296 (encoding a putative glutathione *S*-transferase) [41] and *lrgB* [6] (Fig. 3c and Table S4). Note that *comD* expression was also upregulated in the *lrgAB* strain, although the fold-change (1.3-fold) was lower than the ≥2.0-fold cutoff. Therefore, it is possible that *comDE* is responsible for the upregulation of bacteriocin genes observed in both the wild-type and *lrgAB* mutant during aerobic growth (Table S4). Furthermore, upregulated expression of several genes encoding carbohydrate transporters (PTSs) was observed. These functional categories were confirmed by DAVID functional annotation clustering (Table 1), which identified two significant clusters related to secondary metabolite biosynthesis and carbohydrate transport/PTS systems. SMU_1339–SMU_1342 are located on GI TnSMu2 and encode components of a non-ribosomal peptide (NRP) and polyketide (PK) biosynthesis pathway previously shown to confer tolerance to both aerobic growth and hydrogen peroxide stress [40]. ComDE encodes a two-component regulatory system (TCS) which responds to elevated concentrations of self-produced extracellular competence-stimulating peptide (CSP) (reviewed in [43, 44]). Although the exact link between the Com system and oxidative stress resistance has not been elucidated, expression of this quorum-sensing system has been previously shown to be upregulated during *S. mutans* aerobic growth [22]. *S. mutans* is one of a few Gram-positive bacteria that can both synthesize intracellular glutathione as well as import extracellular glutathione [45, 46]. This small molecule performs a variety of physiological functions, including protection against oxidative stress and cellular redox homeostasis [47], and mutation of the ability of *S. mutans* to synthesize glutathione [47] and reduce oxidized glutathione [42] compromises its ability to withstand oxidative stress. Likewise, expression of the genes encoding thiol peroxidase (Tpx) [48, 49] and LrgA/B [22] has been previously shown to be induced during aerobic growth and/or under conditions of oxidative stress. As described above, mutation of *lrgAB* also has a profound effect on the ability of *S. mutans* to grow aerobically or in the presence of oxidative stress (Fig. 1a; [6]). The fact that altered expression of one or more of these genes is lost in the *lrgAB* mutant is therefore possibly related to its inability to grow under aerobic or oxidative stress.

A large number of upregulated gene expression changes unique to the *lrgAB* mutant were also observed during aerobic growth, including genes belonging to GI TnSMu1 (SMU_199c–SMU_217c), CRISPR2-Cas (SMU_1753 c-55c, SMU_1757c, SMU_1758c, SMU_1760 c-64c) as well as many genes associated with carbohydrate metabolism, ABC transporters and extrachromosomal elements (transposons, prophage) (Fig. 3c and Table S6). DAVID functional clustering analysis confirmed enrichment of upregulated genes involved in carbohydrate transport/PTS systems in the *lrgAB* mutant during aerobic growth (Table 1). Our recent RNA-seq analyses of *S. mutans* UA159 and an isogenic *cidB* mutant also revealed a similar pattern of upregulated gene expression in the *cidB* mutant during aerobic growth, including upregulated expression of TnSmu1 genes [5]. This shared pattern of aerobic gene expression between the *cidB* and *lrgAB* mutants may be significant, given that both mutants have an impaired ability to grow under aerobic and/or oxidative stress conditions [6]. TnSMu1 encodes a variety of putative extrachromosomal elements [50] and although the significance of its upregulation in the *lrgAB* mutant is not clear, many TnSmu1 genes were also upregulated in an *S. mutans clpP* mutant, which has impaired growth in the presence of heat stress [25, 51]. CRISPR-Cas genome editing systems confer adaptive bacterial immunity to phage infection [reviewed in 52], and *S. mutans* contains two CRISPR-Cas loci that function in this manner [53]. Upregulated expression of CRISPR2-associated *cas* genes in response to aerobic growth was also previously observed in an *S. mutans cidB* mutant [5], implying a potential functional link between the Cid/Lrg system and CRISPR-Cas. As suggested previously [5], it is also possible that perturbation of CidB and LrgAB mimic a phage-induced stress response in the cell which activates expression of CRISPR2-Cas, given the predicted similarity of CidA and LrgA to bacteriophage holins. Moreover, mutations in CRISPR1-*cas* and CRISPR2-*cas* in *S. mutans* were both previously shown to confer increased sensitivity to heat stress, as well as decreased resistance to oxidative stress in the case of a CRISPR1-*cas* mutant [24]. Furthermore, a *clpP* mutant (also impaired in its general stress response) was shown to display increased expression of CRISPR2-*cas* genes [25]. Unique patterns of downregulated gene expression in the *lrgAB* mutant during aerobic growth included several ribosomal/translation proteins, ABC transporters and enzymes involved in amino acid metabolism (Fig. 3c and Table S6).

### Gene expression patterns common to wild-type and *lrgAB* mutant in response to heat stress

**Fig. 4.**
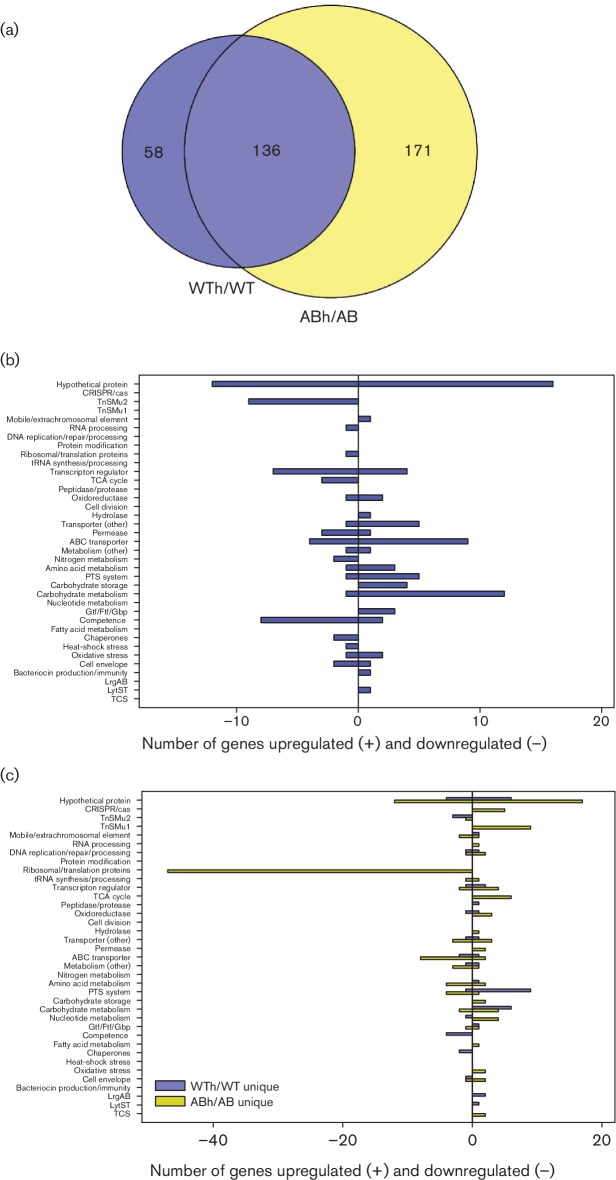
Comparison of wild-type and *lrgAB* mutant gene expression during heat stress growth. (a) Venn diagrams were used to identify overlapping and unique genes in WTh/WT and ABh/AB DE analyses (≥2.0-fold change and *P*<0.005). (b) Functional categorization of 136 overlapping genes between WTh/WT and ABh/AB DE analyses (≥2.0-fold change and *P*<0.005). (c) Functional categorization of 58 and 171 genes unique to WTh/WT (blue bars) and ABh/AB (yellow bars) DE analyses, respectively (≥2.0-fold change and *P*<0.005). For (b) and (c), functional categories were assigned using genomic functional annotations as well as those found in the DAVID and PATRIC databases. Upregulated genes in each category are found to the right of the *y*-axis (positive numbers), and downregulated genes are found to the left of the *y*-axis (negative numbers).

A Venn diagram analysis was also used to differentiate gene expression changes representing the *S. mutans* general response to heat stress from gene expression changes unique to each of the wild-type and *lrgAB* mutant during heat stress (Fig. 4a). Similar to what was observed during aerobic growth, a very large number of DE gene changes were also associated with heat stress in both the wild-type and *lrgAB* mutant. Therefore, the heat stress bioinformatics analyses were performed using a ≥2.0-fold expression change cutoff. This approach revealed that a subset of 136 gene expression changes was common to both WTh/WT and ABh/AB DE analyses (Fig. 4a), with 73 genes upregulated and 63 genes downregulated (Table S7). Functional categories upregulated in response to heat stress were very similar to those upregulated during aerobic growth, whereas expression of TnSMu2 genes and competence genes was uniquely downregulated under this growth condition (Fig. 4b). DAVID functional clustering analysis confirmed significant enrichment of downregulated TnSMu2 genes involved in secondary metabolite biosynthesis, as well as upregulated genes related to starch/sucrose metabolism (Table 1). Surprisingly, expression of chaperone heat-stress-related genes (*groEL*, *grpE*, *groES*), previously shown to be upregulated in response to 42 °C heat shock [54], were downregulated in this current RNA-seq study (Table S7). This discrepancy could be due to differences in heat stress temperature between the two studies (40 °C vs 42 °C), growth condition (rapid heat shock vs continuous growth at higher temperature) and/or sensitivity of transcriptome detection methods used (RNA microarray/qRT-PCR vs RNA-seq). However, other gene expression patterns were conserved, including downregulation of TnSMu2 and competence-related genes [54]. Specifically, downregulated expression of late competence genes during heat stress included *comEA*, *comF*, *comYB*, *comYC* and *comX1*, the regulator of the late competence genes and encoding an alternative sigma factor (Table S7). It is also noteworthy that several genes encoding the agmatine deiminase system (AgDS; *aguBDAC*) were the most highly upregulated (>22-fold in both the wild-type and *lrgAB* mutant) in response to heat stress (Table S7). When agmatine, a decarboxylated derivative of arginine, is catabolized by AgDS, it generates NH_3_, ultimately increasing competitive fitness of *S. mutans* at low pH [55, 56]. Consistent with our result, Griswold *et al.* also showed that AgDS is induced by low pH and heat [56], further supporting the role of AgDS as a general stress response pathway of the organism.

### Gene expression patterns unique to wild-type and *lrgAB* mutant strains in response to heat stress

In total, altered expression of 58 DE genes (35 upregulated, 23 downregulated) was found to be unique to the WTh/WT DE analysis (Fig. 4a and Table S8). Predominant functional categories observed in this subset included upregulated expression of genes encoding hypothetical proteins, PTSs and carbohydrate metabolism, and downregulated expression of hypothetical proteins, late competence proteins and TnSMu2 genes (Fig. 4c). DAVID functional clustering analysis of these 58 genes did not reveal any significantly enriched clusters. Interestingly, *lytS*, *lrgA* and *lrgB* expression were all upregulated in the wild-type strain in response to heat stress (Table S8). Additionally, expression of the heat-shock-related genes *dnaJ* and *dnaK* (encoding chaperone proteins) was downregulated in the wild-type strain in response to heat stress, whereas previous *S. mutans* studies have observed increased expression of *dnaK* in response to heat shock [57, 58].

In contrast to the relatively small number of unique DE genes in the wild-type strain in response to heat stress, 171 unique DE genes (78 upregulated, 93 downregulated) were observed in the *lrgAB* mutant (Table S9). Major functional categories of upregulated genes included hypothetical proteins, CRISPR-Cas, TnSMu1 and TCA cycle enzymes, whereas downregulated functional categories included hypothetical proteins, ribosome/translation proteins, ABC transporters and PTSs (Fig. 4c). By far the largest functional category of DE genes in the *lrgAB* mutant was ribosome/translation proteins, which contained >40 downregulated genes. This was also reflected by DAVID functional annotation clustering, in which ribosome/RNA-binding/translation was the single significantly enriched cluster in the DE genes unique to the *lrgAB* mutant during heat stress growth (Table 1). This result suggests that protein translation may be severely compromised in the *lrgAB* mutant during heat stress. Other heat stress DE genes of interest unique to the *lrgAB* mutant included a number of genes involved in citrate metabolism (upregulated), the VicKR TCS (upregulated), *gtfC* (upregulated), *gtfD* (downregulated), and genes involved in metabolism and/or transport of cystine and glutamine/glutamate (downregulated) (Table S9). VicKR modulates a number of *S. mutans* virulence properties (biofilm formation, bacteriocin production, competence) [59, 60], and, more recently, a *vicK* mutant displayed decreased expression of both *lrgAB* and *lytS* in late exponential-phase cultures [5]. It is therefore interesting that mutation of *lrgAB* has an effect on *vicKR* expression, which may contribute to the altered expression of *gtf* genes also observed in the *lrgAB* mutant during heat stress.

### Gene expression patterns in wild-type and *lrgAB* mutant in response to vancomycin stress

**Fig. 5.**
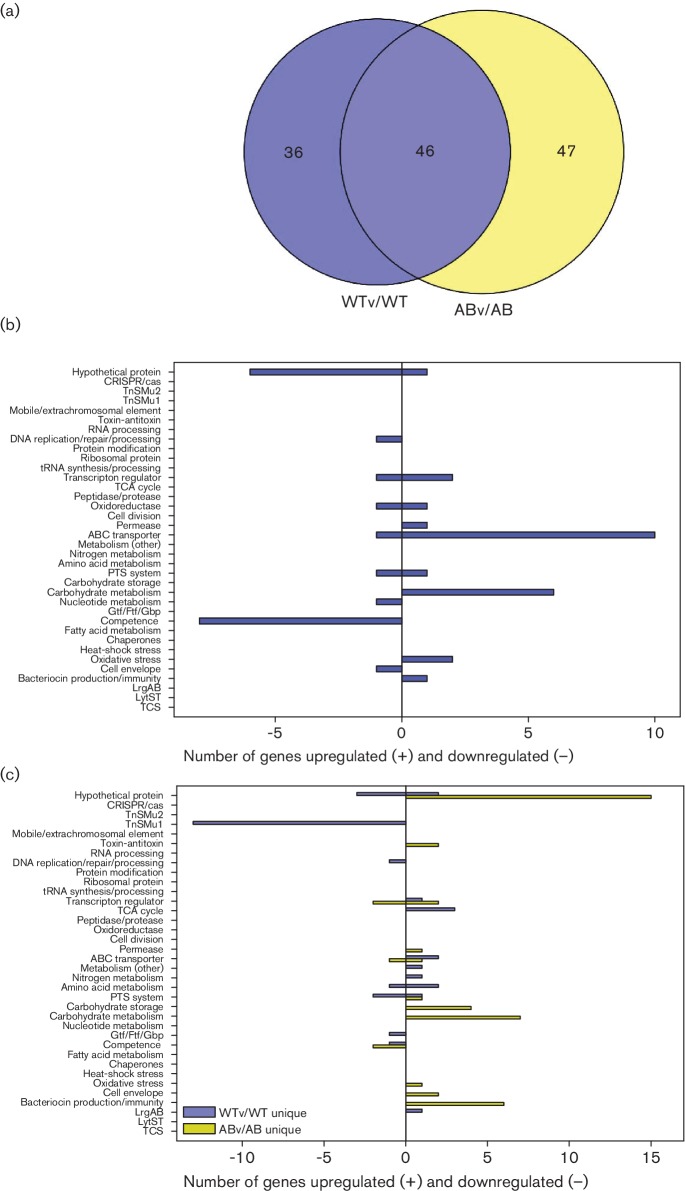
Comparison of wild-type and *lrgAB* mutant gene expression during vancomycin growth. (a) Venn diagrams were used to identify overlapping and unique genes in WTv/WT and ABv/AB DE analyses (≥1.5-fold change and *P*<0.005). (b) Functional categorization of overlapping genes between WTv/WT and ABv/AB DE analyses (≥1.5-fold change and *P*<0.005). (c) Functional categorization of 12 and 15 genes unique to WTv/WT (blue bars) and ABv/AB (yellow bars) DE analyses, respectively (≥1.5-fold change and *P*<0.005). For (b) and (c), functional categories were assigned using genomic functional annotations as well as those found in the DAVID and PATRIC databases. Upregulated genes in each category are found to the right of the *y*-axis (positive numbers), and downregulated genes are found to the left of the *y*-axis (negative numbers).

In general, many fewer gene expression changes were observed between the wild-type and *lrgAB* mutant transcriptomes during vancomycin stress, relative to the aerobic and heat stress conditions. Although the reasons for this are not completely clear, our unpublished proteomics data suggest that a much greater number of changes in proteins were observed when *S. mutans* was grown in the presence of vancomycin. Therefore, it is possible that vancomycin treatment has a more pronounced effect on protein stability/turnover. Given that very few overlapped DE genes between the wild-type and *lrgAB* mutant were identified during vancomycin growth when applying a 2.0-fold expression change cutoff to the data (Fig. S5), the vancomycin gene expression data were instead analysed and presented here using an expression fold-change of ≥1.5 (Fig. 5). Using this criterion, Venn comparison of ABv/AB and WTv/WT DE analyses revealed 46 gene expression changes (25 upregulated, 21 downregulated) common to both the wild-type and *lrgAB* mutant during vancomycin growth (Fig. 5a and Table S10). The majority of upregulated overlapping gene expression changes belonged to ABC transporter and carbohydrate metabolism functional categories, whereas most downregulated expression changes were found to be in genes encoding hypothetical proteins and competence-related genes (Fig. 5b). DAVID functional annotation clustering of the overlapped genes yielded pyruvate metabolism/glycolysis (SMU_1421–SMU_1423, encoding pyruvate dehydrogenase; *pdh*) as the only significantly enriched GO term cluster (Table 1). Upregulated expression changes unique to the wild-type strain included TCA cycle genes (aconitase, citrate synthase, isocitrate dehydrogenase) and genes involved in manganese transport (*sloR*, *sloB*, *sloC*), while downregulated genes included primarily hypothetical and competence-related proteins (Fig. 5c and Table S11). SloR is a DtxR-like transcriptional regulator that represses expression of the *sloABC* operon (encoding a manganese transporter) when manganese concentration is high in the environment [61]. Interestingly, expression of *pdh*, *tpx*, *citZ* and the *glg* operon (genes differentially expressed in the wild-type and/or *lrgAB* mutant during vancomycin stress) have also been shown to be subject to repression by SloR [62]. DAVID functional annotation clustering revealed only one significantly enriched GO term cluster (TCA cycle; Table 1).

Additionally, 47 DE genes (41 upregulated, six downregulated) were found to be unique to the *lrgAB* mutant during vancomycin stress (Fig. 5a and Table S12). Upregulated unique gene expression functional categories included hypothetical proteins, ABC transporters, toxin/antitoxin (T/A) systems, carbohydrate metabolism/storage and bacteriocin-related genes (Fig. 5c). The MazF family of toxins inhibit cell translation and growth by cleaving mRNAs at specific recognition sequences in response to a variety of stress conditions (reviewed in [63]). T/A modules such as MazEF have been suggested to be mediators of bacterial programmed cell death [64, 65], and, interestingly, expression of *mazEF* was also upregulated during aerobic growth in an *S. mutans cidB* mutant [5]. T/A modules (including MazEF) have also been recently implicated in the ability of *S. mutans* biofilm cells to undergo a bimodal response to competence-related quorum sensing signals [66]. The potential functional linkage of T/A modules to the Cid/Lrg system and its role in preserving cellular homeostasis in the face of adverse environmental conditions is currently under investigation by our research group. Downregulated functional categories observed in the *lrgAB* mutant included transcriptional regulators, ABC transporters and late competence genes (Fig. 5c). DAVID functional annotation clustering revealed two significant GO term enrichments for gene expression changes unique to the *lrgAB* mutant during vancomycin stress: lactose/galactose metabolism and starch/sucrose metabolism (Table 1).

### Identification of DE genes common to all three stress conditions for both wild-type and *lrgAB* mutant strains

**Fig. 6.**
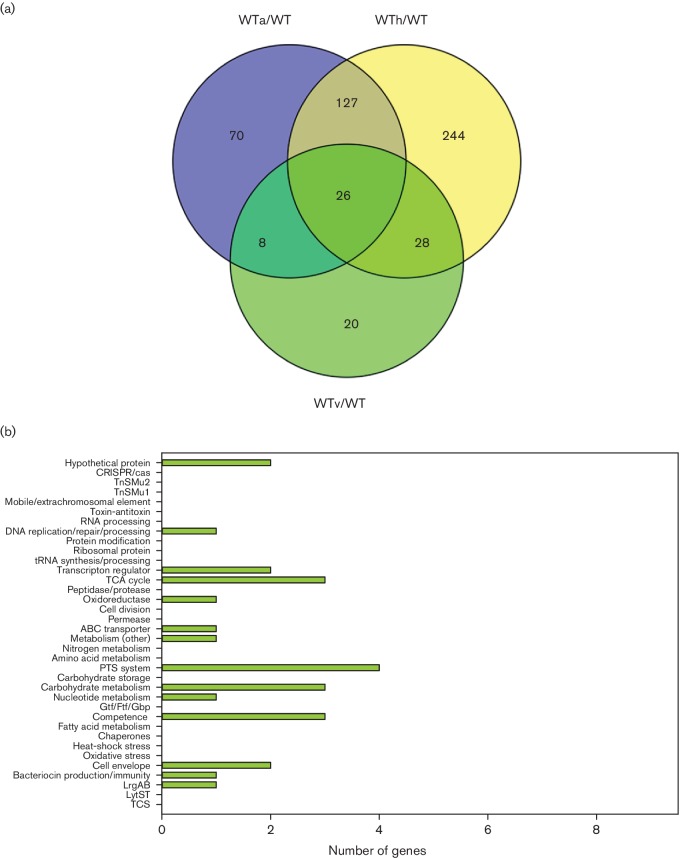
Venn diagrams of aerobic, heat stress and vancomycin DE comparisons for wild-type (a) and corresponding functional categories of the overlapping genes (b). DE genes with ≥1.5-fold change and *P*<0.005 are included in this analysis.

**Fig. 7.**
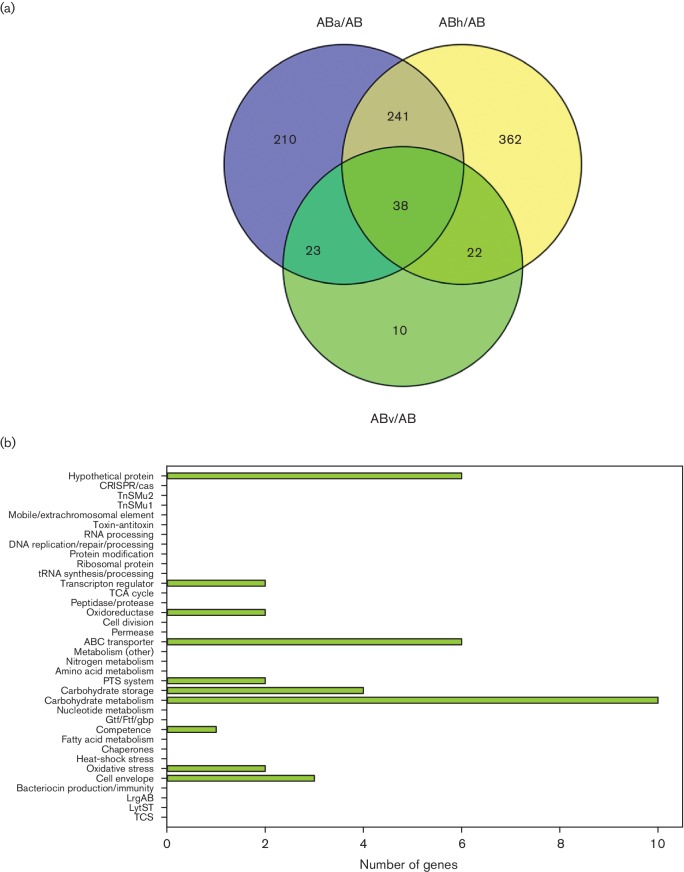
Venn diagrams of aerobic, heat stress and vancomycin DE comparisons for *lrgAB* mutant (a) and corresponding functional categories of the overlapping genes (b). DE genes with ≥1.5 fold change and *P*<0.005 are included in this analysis.

To attempt to identify a core stress regulon common to all three stress conditions (aerobic, heat, vancomycin), the degree of overlap in DE genes existing between all WTx/WT comparisons and all ABx/AB comparisons (where x refers to aerobic, heat stress or vancomycin, as appropriate) was also determined using Venn diagrams (Figs 6 and 7). Because so few DE genes fell within the 2.0-fold expression change cutoff for the vancomycin stress condition, this analysis was performed using a 1.5-fold expression change cutoff for the DE genes in all three stress conditions. Using this less stringent cutoff criterion, 26 DE genes were common to all three stress conditions in the wild-type strain (Fig. 6a) and 38 DE genes were common to all three stress conditions in the *lrgAB* mutant (Fig. 7a). Of these core sets of DE genes, 12 occurred in both the wild-type and *lrgAB* mutant: SMU_1419–23 (encoding, in order, a putative transcriptional regulator, putative oxidoreductase and the PdhABC pyruvate dehydrogenase subunits), SMU_1584c (encoding a putative 67-kDa myosin-cross reactive streptococcal antigen-like protein), SMU_2133c (encoding a membrane protein of unknown function), SMU_114 (fructose permease), SMU_1400c (uncharacterized protein), SMU_1596 (PtcC permease subunit), SMU_1599 (CelR transcriptional regulator) and SMU_1927 (ABC transporter gene). Interestingly, expression of the *pdh* operon was highly expressed in a subpopulation when *S. mutans* experiences sugar starvation [67]. Perhaps upregulated expression of the *pdh* operon (which occurred under all three stress conditions in both the wild-type and *lrgAB* mutant strain) represents a general growth adaptation to suboptimal conditions.

In the wild-type strain, the gene expression changes common under all three stress conditions fell into a number of functional categories (Fig. 6b and Table S13), the largest of which included the PTS, TCA cycle, competence and carbohydrate metabolism. The potential importance of carbohydrate metabolism to the *S. mutans* stress response was reinforced by DAVID analysis of this overlapping gene set in the wild-type strain, as two significantly enriched functional annotation clusters were identified that both related to the TCA cycle/glycolysis (*citB*, *citZ*, *idh*, *pdhABC*). Of additional note is the fact that *lrgB* was induced in the wild-type under all three conditions (4.9-, 2.9- and 2.3-fold under aerobic, heat and vancomycin stress, respectively). Expression of *lrgA* was also upregulated in the wild-type strain by 4.3-fold during aerobic stress, 4.0-fold during heat stress and 1.49-fold under vancomycin stress (just below the 1.5-fold cutoff used for bioinformatics analysis). The fact that *lrgAB* expression was increased under all three stress conditions in the wild-type strain reinforces the previously hypothesized role for the Cid/Lrg system in adapting to stress and/or promoting cell homeostasis.

DAVID analysis of this *lrgAB* mutant overlapping gene set revealed significant enrichment of five functional annotation clusters, the bulk of which were related to carbohydrate metabolism/storage, cell envelope and oxidation–reduction reactions (Table 1). Some of the gene expression changes shared between all stress conditions in the *lrgAB* mutant also followed a similar but accentuated pattern of functional categories (Fig. 7b) relative to that observed in the wild-type strain (Fig. 6b). Specifically, a greater number of genes belonging to hypothetical proteins, ABC transporters and carbohydrate metabolism were observed to be altered in the *lrgAB* mutant compared to the wild-type strain. Additionally, altered expression of carbohydrate storage genes (i.e. *glg* operon), oxidative stress genes (i.e. alkyl hydroperoxide reductase AhpD and associated putative dehydrogenase), SMU_933–SMU_936 (potential l-cystine ABC transporter) and SMU_1603 (putative lactoylglutathione lyase; upregulated under all stress conditions) were uniquely observed in the *lrgAB* mutant strain under all three stress conditions (Table S14). Interestingly, *S. mutans* LguL has been previously shown to be involved in the detoxification of methylglyoxal, a highly toxic glycolytic by-product that can react with a variety of cellular targets [68].

## Conclusions

In general, this RNA-seq approach has confirmed previously reported patterns of *S. mutans* gene expression changes in response to aerobic [22] and heat stress [54], and has identified for the first time a vancomycin stress transcriptome characterized by primarily upregulated expression of genes involved in pyruvate metabolism and ABC transporters. The *lrgAB* growth defect under aerobic and heat stress may be related to altered TnSMu1 and CRISPR-Cas systems, as these were both upregulated in the *lrgAB* mutant under these two stress conditions. The ability of the *lrgAB* mutant to grow under aerobic stress may be additionally compromised by loss of induction of thiol peroxidase and/or glutathione metabolism genes (glutathione reductase, glutathione *S*-transferase), genes which were uniquely upregulated in the wild-type strain during aerobic growth. Furthermore, decreased translation may contribute to the impaired ability of the *lrgAB* mutant to grow well during heat stress, as decreased expression of >40 ribosome/translation proteins was observed in the *lrgAB* mutant. Analysis of the overlapping genes altered in all three stress conditions has also yielded valuable clues to a possible core stress regulon involving regulation and expression of pyruvate dehydrogenase that is shared between the wild-type and *lrgAB* mutant strains. A likely role for LrgAB in contributing to the *S. mutans* general stress response was also suggested by upregulated expression of *lrgAB* in the wild-type strain during aerobic, heat and vancomycin growth, reinforcing the idea that the Cid/Lrg system contributes to cell homeostasis in the face of exogenous stress. Furthermore, a much greater number of genes involved in carbohydrate metabolism/storage, ABC transporters and oxidative stress were altered in the *lrgAB* mutant under all three stress conditions, suggesting that LrgAB is strongly linked to these important cellular processes. However, follow-up experimentation will need to be done to determine whether LrgAB directly or indirectly regulates these aspects of *S. mutans* physiology. Additionally, it is very interesting that the *cidB* and *lrgAB* mutants have a similar transcriptomic response during anaerobic or aerobic growth, suggesting that Cid and Lrg may function as a redundant system to transmit the environmental signals into the regulatory network that modulates homeostasis and virulence. Also, the data provide an important basis for how the *cid* an *lrg* operons, or their gene products, are functionally and mechanically connected. Taken together, the results provide a comprehensive transcriptome database to the *S. mutans* community, which can be used to develop new hypotheses and guide future studies of the *S. mutans* stress response.

## Data bibliography

1. Ajdic D, McShan WM, McLaughlin RE, Savic G, Chang J *et al*. *S. mutans* UA159 NCBI reference sequence: NC_004350.2 (2002).

## Supplementary Data

2582 Supplementary File 1
