# Supplementary material for: Modification of the Streptococcus mutans transcriptome by LrgAB and environmental stressors
Source: Microb Genom. 2017 Feb 28;3(2):e000104. doi: 10.1099/mgen.0.000104 (PMC5361627; doi:10.1099/mgen.0.000104)
Supplement: Supplementary File 1 [file mgen-3-104-s001.pdf]

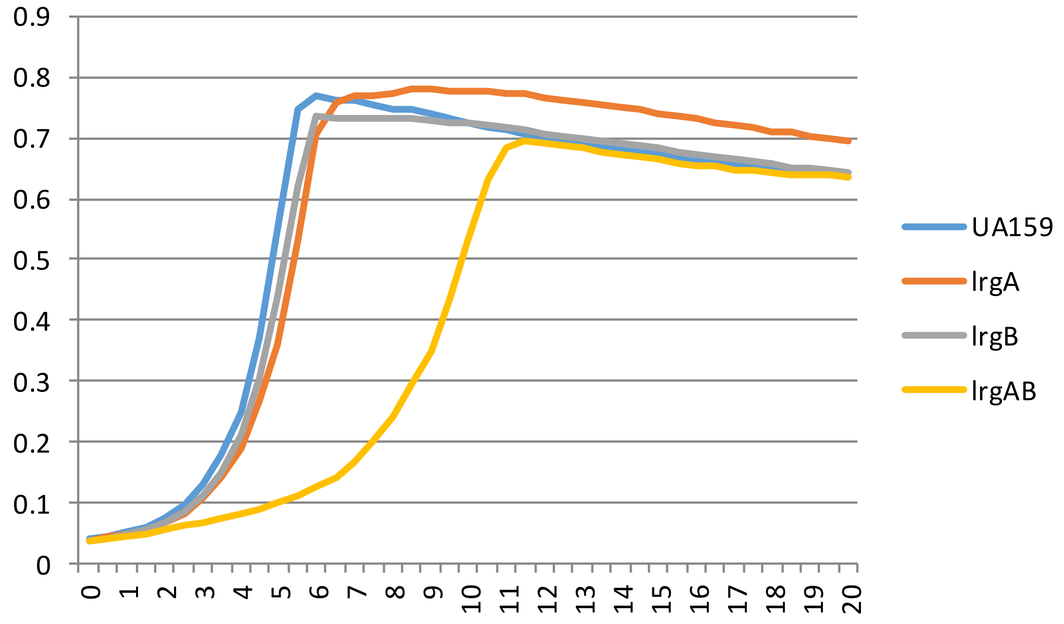


**Supplemental Fig. S1. Growth of *S. mutans* wild-type and isogenic *lrg* mutants at 40°C (anaerobic)**. Growth curves were obtained in a Bioscreen C, as described in Materials and Methods.

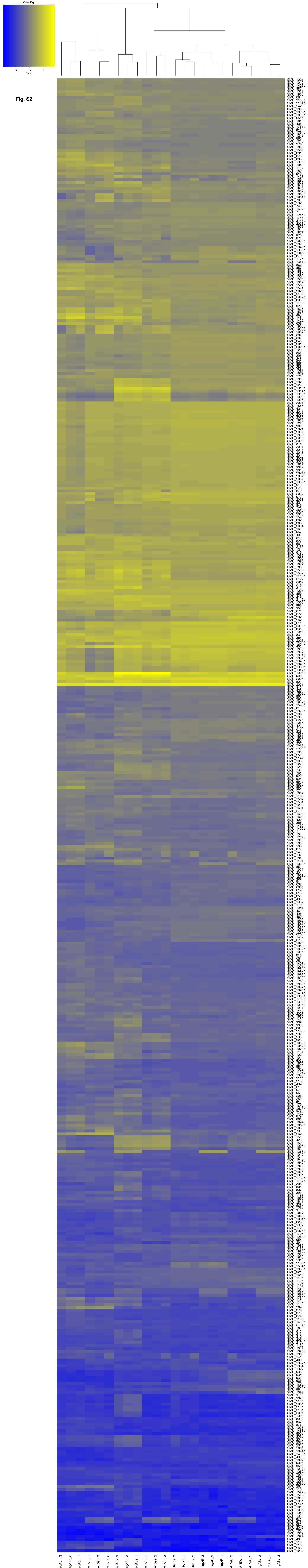

**Fig. S2**

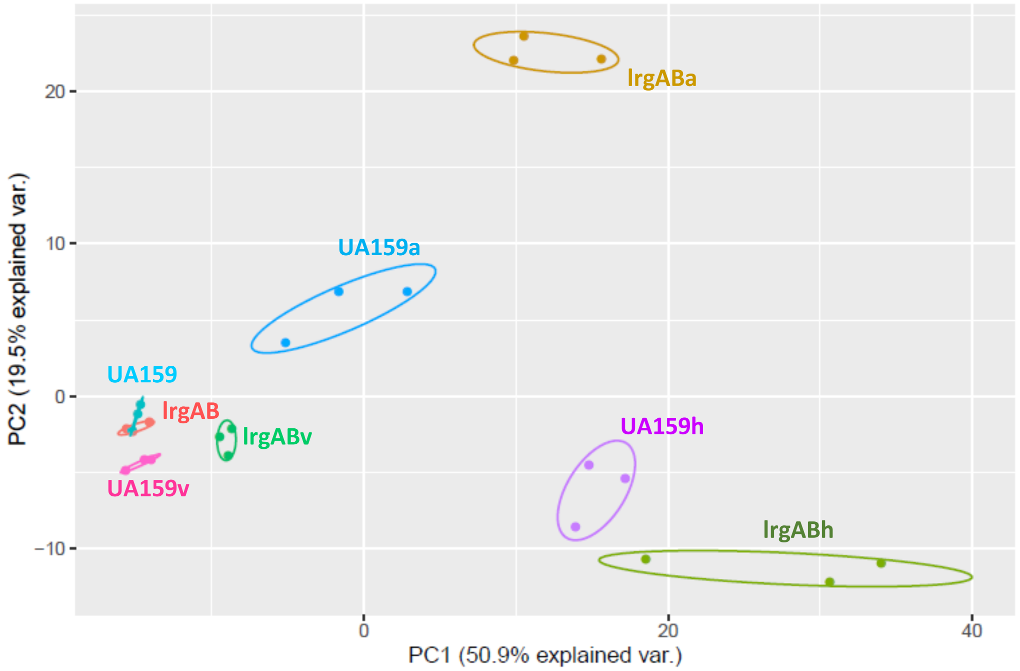


**Supplemental Fig. S3. PCA of RNA-seq data**. DE genes from all 10 pairwise comparisons with > 2-fold change and *P*-value < 0.005 were analyzed by Principle Components Analysis (PCA), as described in Materials and Methods

Fig. S4

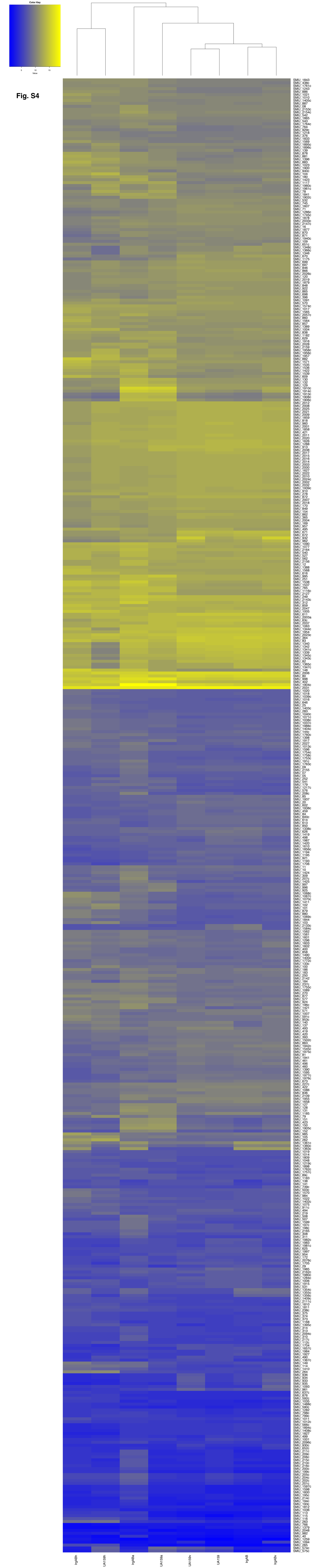

**Fig. S5: Vancomycin at 2-fold cutoff**

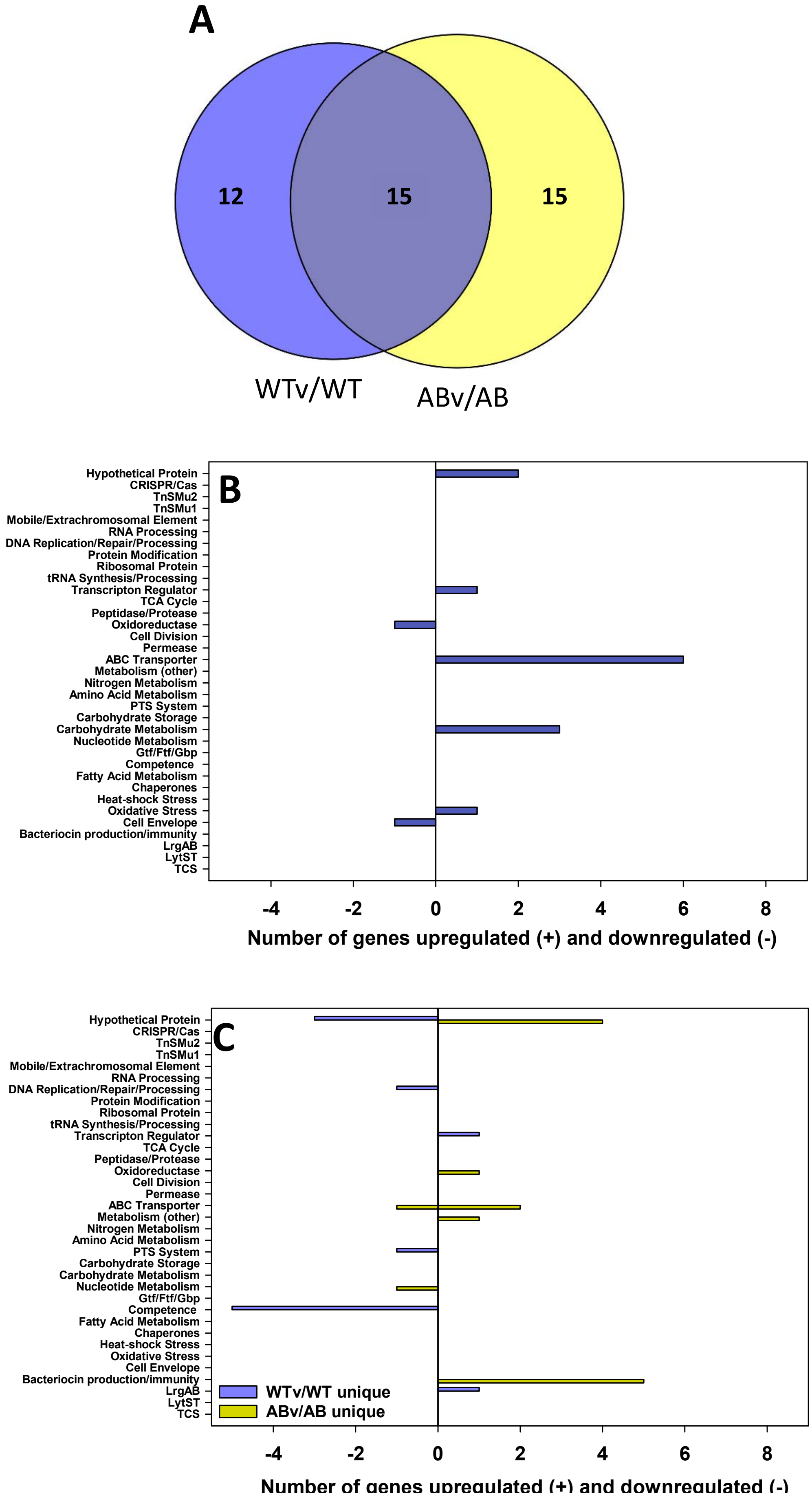

**Table S1.** Oligonucleotide promers used for qRT-PCR in this study

| | Gene | Sense primer | Antisense primer | Product size (bp) |
|---|---|---|---|---|
| SMU_41 | | ATGTTTATAAGAAGTAAGCTGAGAAGG | TCAGTAATTTGTCAAGAAGGAAATG | 85 |
| SMU_82 | *dnaK* | TATCGCTAATCCCGAAGGAA | GACGTTTAGCAGCATCACCA | 89 |
| SMU_191c | | TCCACGGATTAGAAAAGACCA | TGCCTCTCGCTTGGTATTAAA | 109 |
| SMU_423 | *nlmD* | ACAGCTGGTTCTGCAGGTTT | TCCTAAAGCCGCTCCAGATA | 96 |
| SMU_490 | *pflC* | GGTTGTCCTCTACGCTGTCC | AATTTTGTTTCGGCATCCAG | 86 |
| SMU_508 | | CATCAGGTAGACGCCCATTT | CAATGGGATTGGCTCCTAAG | 139 |
| SMU_609 | | TCAGATAAGGCAGCAGCTCA | TGGATTTTGCACCTTTGTCA | 118 |
| SMU_925 | *cipI* | CGTGTTTTTGCTTTTGATGG | CAGCAGCAGCTGAGAAGATG | 89 |
| SMU_940c | *patB* | GGCGAAGAGGTTGCTAACAG | CAGCGGCTATTCCGTAATGT | 109 |
| SMU_1114 | *gyrA* | CCA AGA ATC TGC TGT CCG | TTG CGA CTA TCT GCT ATG TG | 111 |
| SMU_1363c | *tpn* | ATTGTTGGTCAGCCTTCCAC | TAATAGCCCTCTGCCCAAAA | 144 |
| SMU_1396 | *gbpC* | GCTTTTGGAGCAACGAATGT | CCGGTTCTGATGCTTGTGTA | 100 |
| SMU_1421 | *pdhC* | TTGAAGCTCCAGAAGCTGGT | TTGACCAATCCAAGCAATGA | 92 |
| SMU_1752c | | GTTCGTCCATGAAAGCAACA | ATCCACCTGCTGAAAGACCA | 102 |
| SMU_1914c | *cipB* | TTGTGCAGCAGGTATTGCTC | AAGAGCTCCTCCGATTCCTC | 133 |

**Table S2**. Real-tme PCR validation of RNA-Seq results

| | | lrgAB /WT | lrgABa /WTa | lrgABh /WTh | lrgABv /WTv | WTa /WT | WTh /WT | WTv /WT | lrgABa /lrgAB | lrgABh /lrgAB | lrgABv /lrgAB |
|---|---|---|---|---|---|---|---|---|---|---|---|
| | | | | | | | | | | | |
| **SMU_1396** | **Real-time pcr** | 1.2 † | 1.3 | 0.5 | 2.2 | 1.2 | 7.5 | 0.7 | 1.3 | 3.3 | 1.4 |
| | **RNA-Seq** | 1.2 | 1.6 | 1.2 | 2.1 | 1.7 | 5.1 | 0.7 | 2.1 | 5.0 | 1.3 |
| | | | | | | | | | | | |
| **SMU_490** | **Real-time pcr** | 0.5 | 1.3 | 0.2 | 0.5 | 0.3 | 1.2 | 1.2 | 0.7 | 0.4 | 1.1 |
| | **RNA-Seq** | 0.6 | 1.3 | 0.3 | 0.5 | 0.5 | 0.9 | 1.3 | **1.25 *** | 0.5 | 1.0 |
| | | | | | | | | | | | |
| **SMU_41** | **Real-time pcr** | 1.0 | 5.2 | 1.1 | 0.8 | 1.0 | 1.3 | 1.4 | 4.8 | 1.3 | 1.1 |
| | **RNA-Seq** | 2.7 | 5.1 | 0.8 | 0.9 | 4.2 | 3.1 | 3.3 | 7.7 | 0.9 | 1.1 |
| | | | | | | | | | | | |
| **SMU_191c** | **Real-time pcr** | 0.7 | 3.8 | 0.5 | 0.9 | 0.4 | 1.7 | 0.8 | 2.5 | 1.2 | 1.1 |
| | **RNA-Seq** | 0.8 | 3.9 | 0.9 | 0.9 | 0.9 | 1.4 | 0.8 | 4.2 | 1.6 | 0.9 |
| | | | | | | | | | | | |
| **SMU.423** | **Real-time pcr** | 0.7 | 0.7 | 0.6 | 1.2 | 7.5 | 1.0 | 1.1 | 7.5 | 0.9 | 1.9 |
| | **RNA-Seq** | 0.7 | 0.8 | 1.3 | 1.1 | 14.2 | 0.7 | 1.2 | 16.3 | 1.3 | 2.0 |
| | | | | | | | | | | | |
| **SMU.1363c** | **Real-time pcr** | 48.3 | 33.7 | 11.2 | 41.2 | 0.6 | 2.0 | 1.1 | 0.4 | 0.5 | 0.9 |
| | **RNA-Seq** | 37.9 | 31.6 | 17.3 | 31.6 | 1.1 | 1.5 | 0.8 | 0.9 | 0.7 | 0.9 |
| | | | | | | | | | | | |
| **SMU_1421** | **Real-time pcr** | 1.3 | 3.3 | 0.8 | 1.6 | 2.1 | 7.1 | 2.6 | 5.4 | 4.3 | 3.3 |
| | **RNA-Seq** | 1.2 | 3.4 | 1.4 | 1.6 | 2.9 | 4.0 | 2.3 | 7.8 | 4.7 | 3.0 |
| | | | | | | | | | | | |
| **SMU_82** | **Real-time pcr** | 1.1 | 2.2 | 1.0 | 1.0 | 0.3 | 0.4 | 1.0 | 0.5 | 0.4 | 1.0 |
| | **RNA-Seq** | 1.1 | 2.2 | 2.3 | 1.0 | 0.5 | 0.3 | 1.2 | 0.9 | 0.6 | 1.0 |
| | | | | | | | | | | | |
| **SMU_508** | **Real-time pcr** | 1.0 | 2.4 | 0.8 | 1.6 | 1.1 | 1.2 | 0.9 | 2.8 | 1.0 | 1.4 |
| | **RNA-Seq** | 1.1 | 3.0 | 1.3 | 1.2 | 2.1 | 1.0 | 1.1 | 5.6 | 1.2 | 1.2 |
| | | | | | | | | | | | |
| **SMU_925** | **Real-time pcr** | 1.1 | 0.6 | 0.6 | 1.0 | 3.2 | 1.4 | 1.0 | 1.8 | 0.8 | 0.9 |
| | **RNA-Seq** | 1.0 | 0.6 | 1.4 | 1.1 | 5.0 | 0.7 | 0.8 | 3.1 | 1.0 | 0.9 |
| | | | | | | | | | | | |
| **SMU_940c** | **Real-time pcr** | 1.1 | 1.8 | 0.5 | 1.3 | 0.4 | 3.6 | 1.1 | 0.6 | 1.7 | 1.2 |

| | | | | | | | | | | | |
|---|---|---|---|---|---|---|---|---|---|---|---|
| | **RNA-Seq** | 1.3 | 2.2 | 1.1 | 1.3 | 0.6 | 2.7 | 1.0 | 0.9 | 2.3 | 1.0 |
| | | | | | | | | | | | |
| **SMU_1752c** | **Real-time pcr** | 1.0 | 1.8 | 0.4 | 1.1 | 0.6 | 1.9 | 0.9 | 1.2 | 0.7 | 1.0 |
| | **RNA-Seq** | 0.9 | 2.1 | 0.8 | 1.0 | 1.0 | 1.4 | 0.8 | 2.3 | 1.2 | **0.9** |

† Results are expressed in fold change and normalized by expression of 16S RNA

* Blue Statistically flipped expression

**Table S3.** Altered gene expression comparing *lrgAB* to wild-type during anaerobic (unstressed) growth (≥ 1.5-fold change, $P < 0.005$)

| Gene ID | Fold-change (AB/WT) | Gene | Function |
|---|---|---|---|
| SMU_1363c | 37.92 | *tpn* | transposase fragment (IS605/IS200-like) |
| SMU_1360c | 5.07 | | hypothetical protein |
| SMU_1354c | 4.95 | | transposase |
| SMU_1355c | 4.84 | *tnp* | transposase |
| SMU_1356c | 4.45 | *tpn* | transposase |
| SMU_1361c | 4.41 | *yjjB* | transcriptional regulator |
| SMU_1365c | 2.25 | | Permease-FtsX-like permease |
| SMU_1347c | 2.08 | | Permease-ABC-type antimicrobial peptide transport system, Permease component |
| SMU_1348c | 2.01 | | ABC transporter ATP-binding Protein |
| SMU_1366c | 2.00 | | ABC transporter ATP-binding Protein |
| SMU_654 | 1.76 | | ABC transporter, ATP-binding protein |
| SMU_933 | 1.73 | *atmA* | amino acid ABC transporter, amino acid substrate-binding protein |
| SMU_935 | 1.73 | | amino acid ABC transporter, permease protein |
| SMU_936 | 1.73 | | amino acid ABC transporter, ATP-binding protein |
| SMU_932 | 1.68 | | conserved hypothetical protein |
| SMU_934 | 1.67 | | amino acid ABC transporter, permease protein |
| SMU_184 | 1.61 | *fimA* | ABC transporter element, iron predicted binding protein |
| SMU_2133c | 1.55 | | conserved hypothetical protein (possible transmembrane protein) |
| SMU_961 | 1.54 | | macrophage infectivity potentiator-related protein |
| SMU_962 | 1.54 | *mmgC* | acyl-CoA dehydrogenase |
| SMU_183 | 1.51 | *sloB* | manganese ABC transporter permease element |
| SMU_207c | 0.65 | | transcriptional regulator |
| SMU_195c | 0.64 | | hypothetical protein |
| SMU_152 | 0.63 | | hypothetical protein |
| SMU_208c | 0.63 | | conserved hypothetical protein, FtsK/SpoIIIE family |
| SMU_2042 | 0.62 | *dexT* | dextranase ( 1,6-alpha-glucanhydrolase ) |
| SMU_201c | 0.61 | | conserved hypothetical protein |
| SMU_211c | 0.61 | | hypothetical protein |
| SMU_209c | 0.61 | | hypothetical protein |
| SMU_198c | 0.61 | *tpn* | conjugative transposon protein |
| SMU_197c | 0.60 | | hypothetical protein |
| SMU_206c | 0.60 | | hypothetical protein |
| SMU_150 | 0.60 | *nlmA* | non-lantibiotic mutacin IV A |
| SMU_196c | 0.56 | | immunogenic secreted protein (transfer protein) |
| SMU_490 | 0.55 | *pflC* | pyruvate formate-lyase activating enzyme |
| SMU_200c | 0.54 | | hypothetical protein |

| | | | |
|---|---|---|---|
| SMU_204c | 0.53 | | hypothetical protein |
| SMU_202c | 0.52 | | conserved hypothetical protein/Streptococcus-specific protein |
| SMU_210c | 0.51 | | hypothetical protein |
| SMU_205c | 0.50 | | conserved hypothetical protein |
| SMU_495 | 0.50 | *gldA* | glycerol dehydrogenase |
| SMU_494 | 0.49 | *mipB* | transaldolase family protein |
| SMU_493 | 0.48 | *pfl pfl-2* | formate acetyltransferase (pyruvate-formate lyase) |
| SMU_199c | 0.48 | | hypothetical protein |
| SMU_193c | 0.43 | | conserved hypothetical protein |
| SMU_1367c | 0.42 | | conserved hypothetical protein |
| SMU_141 | 0.40 | | conserved hypothetical protein |
| SMU_139 | 0.37 | *oxdC* | oxalate decarboxylase |
| SMU_140 | 0.35 | *gshR* | glutathione reductase |
| SMU_137 | 0.35 | *mleS* | malolactic enzyme |
| SMU_138 | 0.33 | *mleP* | malate permease/auxin efflux carrier |
| SMU_575c | 0.14 | *lrgA* | murein hydrolase regulator |
| SMU_574c | 0.10 | *lrgB* | effector of murein hydrolase |

**Table S4.** Altered gene expression common to both wild-type and *lrgAB* mutant during aerobic growth (≥ 2-fold change, $P < 0.005$)

| Gene ID | Fold-change (WTa/WT) | Fold-change (ABa/AB) | Gene | Function |
|---|---|---|---|---|
| SMU_423 | 14.2 | 16.3 | *nlmD* | possible bacteriocin |
| SMU_151 | 14.2 | 17.8 | | non-lantibiotic mutacin IV B |
| SMU_1908c | 13.4 | 17.5 | | hypothetical protein |
| SMU_1910c | 13.3 | 17.3 | | hypothetical protein |
| SMU_150 | 13.2 | 17.4 | | non-lantibiotic mutacin IV A |
| SMU_153 | 13.1 | 11.5 | | hypothetical protein |
| SMU_1906c | 13 | 18.3 | | bacteriocin-related protein |
| SMU_1909c | 13 | 17.8 | | hypothetical protein |
| SMU_1912c | 12.9 | 16.8 | | hypothetical protein |
| SMU_1914c | 12.8 | 20.4 | | bacteriocin protein, BlpO-like |
| SMU_1913c | 12.6 | 16.5 | | hypothetical protein; immunity protein, BLpL-like |
| SMU_152 | 12.2 | 12.5 | | hypothetical protein |
| SMU_1905c | 10.9 | 12.1 | | Putative bacteriocin secretion protein |
| SMU_1904c | 10.7 | 11.7 | | hypothetical protein |
| SMU_1903c | 10.6 | 12.5 | | hypothetical protein |
| SMU_1117 | 6.4 | 4.8 | *naoX* | H2O-forming NADH Oxidase |
| SMU_1116c | 5.3 | 4.7 | | hypothetical protein |
| SMU_79 | 5.3 | 2.2 | *fruB* | fructan hydrolase; exo-beta-D-fructosidase |
| SMU_925 | 5.03 | 3.1 | | bacteriocin immunity protein |
| SMU_78 | 5 | 2.7 | *fruA* | fructan hydrolase; exo-beta-D-fructosidase |
| SMU_998 | 4.6 | 3 | | ABC transporter, ferrichrome-binding protein |
| SMU_997 | 4.5 | 3.1 | | inorganic ion ABC transporter,ATP-binding protein; possible ferrichrome transport system |
| SMU_575c | 4.3 | 3.3 | | murein hydrolase regulator |
| SMU_765 | 4.2 | 3.8 | | alkyl hydroperoxide reductase, subunit F |
| SMU_996 | 4.2 | 2.9 | | ABC transporter, permease protein;possible ferrichrome transport system |
| SMU_995 | 3.8 | 2.8 | | ferrichrome ABC transporter (permease) |
| SMU_764 | 3.6 | 3 | *ahpC* | alkyl hydroperoxide reductase, subunit C |
| SMU_131 | 3.53 | 5.4 | *lplA* | lipoate-protein ligase |
| SMU_130 | 3.3 | 5 | *adhD* | dihydrolipoamide dehydrogenase |
| SMU_629 | 3.3 | 2.4 | *sod* | superoxide dismutase |
| SMU_129 | 3.1 | 4.6 | *adhC* | dihydrolipoamide S-acetyltransferase |
| SMU_1902c | 3 | 2.6 | | hypothetical protein |
| SMU_1217c | 3 | 2 | | amino acid ABC transporter, substrate binding protein |
| SMU_1421 | 2.9 | 7.8 | *pdhC* | dihydrolipoamide acetyltransferase (acetoin dehydrogenase E2 component) |
| SMU_132 | 2.8 | 4.5 | | amino acid amidohydrolase (hippurate amidohydrolase) |
| SMU_128 | 2.8 | 3.6 | *adhB* | acetoin dehydrogenase (TPP-dependent) E1 component beta subunit |
| SMU_507 | 2.8 | 6.6 | | transcriptional regulator, DeoR family |
| SMU_1425 | 2.7 | 6.6 | *clpB* | ATP-dependent Clp protease, ATP-binding subunit ClpB |
| SMU_115 | 2.7 | 6.4 | *fruD* | PTS system, fructose-specific IIA component |
| SMU_1536 | 2.6 | 5.2 | *glgA* | glycogen synthase |
| SMU_1537 | 2.6 | 6 | *glgD* | glycogen biosynthesis protein |

| | | | | |
|---|---|---|---|---|
| SMU_104 | 2.5 | 2.1 | | glycosyl hydrolase, alpha-glucosidase |
| SMU_180 | 2.5 | 6.3 | | oxidoreductase, possible fumarate reductase |
| SMU_577 | 2.5 | 3.4 | *lytS* | sensor histidine kinase |
| SMU_541 | 2.5 | 2.7 | | conserved hypothetical protein |
| SMU_1422 | 2.5 | 7.9 | *pdhB* | Putative pyruvate dehydrogenase E1 component beta subunit (EC 1.2.4.1) |
| SMU_1243 | 2.5 | 2.3 | | conserved hypothetical protein (possible low temperature requirement protein A) |
| SMU_1423 | 2.5 | 8.9 | *pdhA* | Putative pyruvate dehydrogenase, TPP-dependent E1 component alpha-subunit (EC 1.2.4.1) |
| SMU_1535 | 2.4 | 4.2 | *phsG* | glycogen phosphorylase |
| SMU_1538 | 2.4 | 6.7 | *glgC* | Glucose-1-phosphate adenylyltransferase (EC 2.7.7.27) (ADP-glucose pyrophosphorylase) (ADPGlc PPase) (ADP-glucose synthase) |
| SMU_114 | 2.4 | 8.2 | | Constitutive fructose permease (Putative PTS system, fructose-specific IIBC component) |
| SMU_2127 | 2.3 | 5 | | Putative succinate semialdehyde dehydrogenase |
| SMU_576 | 2.3 | 3.6 | *lytT* | Sensory transduction protein LytR |
| SMU_1067c | 2.3 | 2.8 | | Putative ABC transporter, permease protein |
| SMU_103 | 2.3 | 2.6 | | Putative PTS system, IIA component |
| SMU_402 | 2.2 | 5.2 | *pfl* | Formate acetyltransferase (EC 2.3.1.54) (Pyruvate formate-lyase) |
| SMU_127 | 2.2 | 2.6 | *adhA* | Putative acetoin dehydrogenase (TPP-dependent), E1 component alpha subunit |
| SMU_148 | 2.2 | 11.8 | *adhE* | Aldehyde-alcohol dehydrogenase |
| SMU_1068c | 2.2 | 3 | | Putative ABC transporter, ATP-binding protein |
| SMU_102 | 2.2 | 2.7 | | Putative PTS system, IID component |
| SMU_1599 | 2.2 | 7.8 | *celR* | Putative transcriptional regulator possible antiterminator |
| SMU_116 | 2.2 | 6.8 | *lacD2* | Tagatose 1,6-diphosphate aldolase 2 (EC 4.1.2.40) (D-tagatose-1,6-bisphosphate aldolase 2) (Tagatose-bisphosphate aldolase 2) |
| SMU_508 | 2.1 | 5.6 | | Uncharacterized protein |
| SMU_1069c | 2.1 | 2.9 | | Uncharacterized protein |
| SMU_881 | 2 | 2.8 | *gtfA* | Sucrose phosphorylase (EC 2.4.1.7) (Glucosyltransferase-A) (GTF-A) (Sucrose glucosyltransferase) |
| SMU_673 | 0.4 | 0.44 | | Uncharacterized protein |
| SMU_1657c | 0.4 | 0.32 | | Putative nitrogen regulatory protein PII |
| SMU_1658 | 0.4 | 0.4 | *nrgA* | Ammonium transporter |
| SMU_670 | 0.33 | 0.32 | *citB* | Aconitate hydratase A (ACN) (Aconitase) (EC 4.2.1.3) ((2R,3S)-2-methylisocitrate dehydratase) ((2S,3R)-3-hydroxybutane-1,2,3-tricarboxylate dehydratase) (Iron-responsive protein-like) (IRP-like) (Probable 2-methyl-cis-aconitate hydratase) (EC 4.2.1.99) (RNA-binding protein) |
| SMU_671 | 0.32 | 0.3 | *citZ* | Citrate synthase (EC 2.3.3.16) |
| SMU_1584c | 0.3 | 0.2 | | Putative 67 kDa myosin-crossreactive streptococcal antigen-like protein |
| SMU_672 | 0.3 | 0.3 | *idh* | Isocitrate dehydrogenase [NADP] (IDH) (EC 1.1.1.42) (IDP) (NADP(+)-specific ICDH) (Oxalosuccinate decarboxylase) |
| SMU_2133c | 0.2 | 0.2 | | Putative membrane protein |

**Table S5.** Altered gene expression unique to wild-type during aerobic growth (≥ 2-folg change, $P < 0.005$)

| Gene ID | Fold-change (WTa/WT) | Gene | Function |
|---|---|---|---|
| SMU_574c | 4.9 | *lrgB* | LrgA-associated membrane protein LrgB |
| SMU_838 | 3.0 | *gshR* | Glutathione reductase (EC 1.8.1.7) |
| SMU_929c | 2.9 | | FIG01118515: hypothetical protein |
| SMU_924 | 2.7 | *tpx* | Thiol peroxidase, Tpx-type (EC 1.11.1.15) |
| SMU_1916 | 2.6 | *comD* | Histidine kinase of the competence regulon ComD |
| SMU_1259 | 2.4 | | type II restriction endonuclease, putative |
| SMU_1296 | 2.4 | | Glutathione S-transferase, Streptococcal type (EC 2.5.1.18) |
| SMU_1865 | 2.3 | *mutY* | A/G-specific adenine glycosylase (EC 3.2.2.-) |
| SMU_1917 | 2.3 | *comE* | Response regulator of the competence regulon ComE |
| SMU_1048 | 2.2 | | Adenylate cyclase |
| SMU_1956c | 2.2 | | PTS system, fructose- and mannose-inducible putative EII component |
| SMU_1957 | 2.2 | | PTS system, fructose- and mannose-inducible IID component (EC 2.7.1.69) |
| SMU_1183 | 2.1 | *mtlF* | PTS system, mannitol-specific IIA component (EC 2.7.1.69) |
| SMU_1958c | 2.1 | | PTS system, fructose- and mannose-inducible IIC component (EC 2.7.1.69) |
| SMU_1182 | 2.1 | *mtlD* | Mannitol-1-phosphate 5-dehydrogenase (EC 1.1.1.17) |
| SMU_105 | 2.1 | | Sucrose operon repressor ScrR, LacI family |
| SMU_1960c | 2.0 | | PTS system, fructose- and mannose-inducible IIB component (EC 2.7.1.69) |
| SMU_1961c | 2.0 | | PTS system, fructose- and mannose-inducible IIA component (EC 2.7.1.69) |
| SMU_1071c | 2.0 | | FIG01116996: hypothetical protein |
| SMU_570 | 0.5 | *feoB* | Ferrous iron transport protein B |
| SMU_571 | 0.5 | | FIG01117138: hypothetical protein |
| SMU_1342 | 0.5 | *bacA1* | Long-chain-fatty-acid--CoA ligase (EC 6.2.1.3) |
| SMU_82 | 0.5 | *dnaK* | Chaperone protein DnaK |
| SMU_1341c | 0.4 | | Long-chain-fatty-acid--CoA ligase (EC 6.2.1.3) |
| SMU_1340 | 0.4 | *bacA2* | putative bacitracin synthetase |
| SMU_1339 | 0.4 | *bacD* | Long-chain-fatty-acid--CoA ligase (EC 6.2.1.3) |
| SMU_29 | 0.3 | *purC* | Phosphoribosylaminoimidazole-succinocarboxamide synthase (EC 6.3.2.6) |

**Table S6**. Altered expression unique to *lrgAB* mutant during aerobic growth (≥ 2-fold change; $P < 0.005$)

| Gene ID | Fold-change (ABa/AB) | Gene | Function |
|---|---|---|---|
| SMU_210c | 14.1 | | hypothetical protein |
| SMU_211c | 11.7 | | hypothetical protein |
| SMU_209c | 9.6 | | hypothetical protein |
| SMU_202c | 9.4 | | conserved hypothetical protein/Streptococcus-specific protein |
| SMU_204c | 9.2 | | hypothetical protein |
| SMU_206c | 8.7 | | hypothetical protein |
| SMU_205c | 8.6 | | conserved hypothetical protein |
| SMU_113 | 8.6 | *pfk* | fructose-1-phosphate kinase |
| SMU_200c | 8.5 | | hypothetical protein |
| SMU_207c | 8.5 | | transcriptional regulator, Cro/CI family |
| SMU_208c | 8.3 | | conserved hypothetical protein, FtsK/SpoIIIE family |
| SMU_216c | 8.2 | | hypothetical protein |
| SMU_199c | 8.1 | | hypothetical protein |
| SMU_41 | 7.7 | | hypothetical protein (protein) |
| SMU_201c | 7.7 | | conserved hypothetical protein |
| SMU_215c | 7.7 | | hypothetical protein |
| SMU_213c | 7.7 | | hypothetical protein |
| SMU_1600 | 6.5 | *celB* | PTS system IIB component, required for cellobiose uptake and metabolism |
| SMU_1539 | 6.2 | *glgB* | 1,4-alpha-glucan branching enzyme |
| SMU_214c | 6.2 | | hypothetical protein |
| SMU_1424 | 6.1 | *acoL adhD* | dihydrolipoamide dehydrogenase |
| SMU_196c | 5.9 | | immunogenic secreted protein (transfer protein) |
| SMU_212c | 5.8 | | hypothetical protein |
| SMU_198c | 5.7 | *tpn* | conjugative transposon protein |
| SMU_197c | 5.7 | | hypothetical protein |
| SMU_40 | 5.7 | | conserved hypothetical protein |
| SMU_1598 | 5.3 | *celC* | PTS system IIA component, required for cellobiose uptake and metabolism |
| SMU_195c | 5.2 | | hypothetical protein |
| SMU_1596 | 5.1 | *celB* | PTS system, cellobiose-specific IIC component |
| SMU_149 | 5.1 | *tpn* | transposase fragment (IS605/IS200-like) |
| SMU_309 | 5.1 | *slrR* | regulator of sorbitol operon |
| SMU_194c | 4.8 | | conserved hypothetical protein, phage-related |
| SMU_1597c | 4.7 | | conserved hypothetical protein |
| SMU_193c | 4.6 | | conserved hypothetical protein |
| SMU_310 | 4.6 | *srlM* | sorbitol operon activator |

| | | | |
|---|---|---|---|
| SMU_311 | 4.5 | *srlA* | PTS system, sorbitol (glucitol) phosphotransferase enzyme IIC2 |
| SMU_217c | 4.5 | | conserved hypothetical protein; Streptococcus-specific protein |
| SMU_308 | 4.5 | *srlD* | sorbitol-6-phosphate 2-dehydrogenase |
| SMU_191c | 4.2 | | phage-related integrase |
| SMU_312 | 4.1 | *srlE* | PTS system, sorbitol phosphotransferase enzyme IIBC |
| SMU_313 | 4.0 | *srlB* | PTS system, sorbitol-specific enzyme IIA |
| SMU_179 | 4.0 | | conserved hypothetical protein (possible oxidoreductase) |
| SMU_1841 | 3.7 | *scrA* | PTS system, sucrose-specific IIABC component |
| SMU_314 | 3.6 | | hypothetical protein |
| SMU_877 | 3.5 | *aga* | alpha-galactosidase (melibiase) |
| SMU_1411 | 3.4 | | conserved hypothetical protein |
| SMU_609 | 3.3 | *bsp* | cell wall protein precursor |
| SMU_878 | 3.2 | *msmE* | ABC transporter, sugar-binding protein |
| SMU_1601 | 3.1 | *celA* | 6-phospho-beta-glucosidase, required for cellobiose uptake and metabolism |
| SMU_2027 | 3.0 | | transcriptional regulator/repressor |
| SMU_1844 | 3.0 | *scrR* | sucrose operon repressor |
| SMU_2028 | 3.0 | *ftf* | fructosyltransferase |
| SMU_1077 | 3.0 | *pgmA* | phosphoglucomutase |
| SMU_1013c | 2.9 | *yxiQ* | Mg2+/citrate complex transporter |
| SMU_1410 | 2.9 | *frdC* | fumarate reductase |
| SMU_879 | 2.9 | *msmF* | ABC transporter, sugar permease protein |
| SMU_880 | 2.8 | *msmG* | multiple sugar-binding transport system permease protein MsmG |
| SMU_07 | 2.8 | *pth* | peptidyl-tRNA hydrolase |
| SMU_05 | 2.8 | | conserved hypothetical protein |
| SMU_2154c | 2.8 | | peptidase, M16 family |
| SMU_08 | 2.7 | *mfd* | transcription-repair coupling factor |
| SMU_883 | 2.6 | *dexB* | glucan 1,6-alpha-glucosidase |
| SMU_09 | 2.6 | | small RNA binding protein |
| SMU_11 | 2.6 | | Streptococcus-specific protein |
| SMU_2057c | 2.6 | *cadA* | cadmium-efflux ATPase, E1-E2 (heavy metal-transporting ATPase) |
| SMU_1753c | 2.6 | | conserved hypothetical protein |
| SMU_1755c | 2.6 | | conserved hypothetical protein |
| SMU_1070c | 2.6 | | conserved hypothetical protein |
| SMU_10 | 2.6 | *divIC* | cell-division protein DivIC |
| SMU_1764c | 2.5 | | conserved hypothetical protein |
| SMU_101 | 2.5 | *sorC* | sorbose PTS system, IIC component |
| SMU_882 | 2.5 | *msmK* | multiple sugar-binding transport ATP-binding protein MsmK |
| SMU_1757c | 2.5 | | conserved hypothetical protein |
| SMU_2153c | 2.5 | | peptidase, M16 family |

| | | | |
|---|---|---|---|
| SMU_1561 | 2.5 | *trkB* | potassium uptake protein B |
| SMU_1089 | 2.5 | | conserved hypothetical protein |
| SMU_1843 | 2.5 | *scrB* | sucrose-6-phosphate hydrolase |
| SMU_1090 | 2.5 | | conserved hypothetical protein |
| SMU_1562 | 2.5 | *trkA* | potassium uptake protein A |
| SMU_1761c | 2.5 | | conserved hypothetical protein |
| SMU_1763c | 2.5 | | conserved hypothetical protein |
| SMU_1762c | 2.5 | | conserved hypothetical protein |
| SMU_1754c | 2.4 | | conserved hypothetical protein |
| SMU_798c | 2.4 | | hypothetical protein |
| SMU_438c | 2.4 | | (R)-2-hydroxyglutaryl-CoA dehydratase activator-related protein |
| SMU_2165 | 2.4 | *smc* | chromosome segregation protein, ParB-like nuclease domain |
| SMU_252 | 2.3 | | hypothetical protein |
| SMU_527 | 2.3 | | conserved hypothetical protein |
| SMU_1760c | 2.3 | | conserved hypothetical protein |
| SMU_264 | 2.3 | *aguA* | agmatine deiminase |
| SMU_1752 | 2.3 | | hypothetical protein |
| SMU_1409c | 2.3 | *bglC* | transcriptional regulator |
| SMU_1758c | 2.3 | | conserved hypothetical protein |
| SMU_2164 | 2.3 | *htrA* | serine protease |
| SMU_237c | 2.2 | *drrB* | ABC transporter multidrug permease protein |
| SMU_1812 | 2.2 | *tpn* | transposase, IS1193n-related |
| SMU_250 | 2.2 | *nifU* | nitrogen fixation-like protein, NifU |
| SMU_12 | 2.2 | | conserved hypothetical protein |
| SMU_1603 | 2.2 | *lguL* | lactoylglutathione lyase |
| SMU_265 | 2.2 | *arcC* | carbamate kinase |
| SMU_2156 | 2.1 | *recF* | recombination protein F (DNA replication and repair ATPase) |
| SMU_2094c | 2.1 | *arcE* | transcriptional regulator |
| SMU_799c | 2.1 | | conserved hypothetical protein |
| SMU_251 | 2.1 | | ABC transporter permease |
| SMU_112c | 2.1 | | transcriptional regulator, RpiR family |
| SMU_249 | 2.1 | *nifS* | class-V aminotransferase, NifS protein homolog, |
| SMU_542 | 2.1 | *glk* | glucose kinase |
| SMU_1396 | 2.1 | *gbpC* | glucan-binding protein C |
| SMU_263 | 2.1 | *aguD* | amino acid permease /putrescine antiporter |
| SMU_543 | 2.0 | | conserved hypothetical protein |
| SMU_2143c | 2.0 | *trmU* | tRNA (5-methylaminomethyl-2-thiouridylate)-methyltransferase |
| SMU_2142 | 2.0 | *rpiA* | sugar-phosphate isomerase (ribose 5-phosphate isomerase) |
| SMU_238c | 2.0 | | ABC transporter, ATP-binding protein |
| SMU_540 | 2.0 | *dpr* | peroxide resistance protein / iron binding protein |

| | | | |
|---|---|---|---|
| SMU_529 | 2.0 | | hypothetical protein |
| SMU_745 | 0.5 | *yniG* | drug-export protein; multidrug resistance protein |
| SMU_850 | 0.5 | | acetyltransferase, GNAT family |
| SMU_393 | 0.5 | | conserved hypothetical protein |
| SMU_697 | 0.5 | *infC* | translation initiation factor IF-3 |
| SMU_933 | 0.5 | *atmA* | amino acid ABC transporter, amino acid substrate-binding protein |
| SMU_532 | 0.5 | *trpE* | anthranilate synthase, component I |
| SMU_278 | 0.5 | | hypothetical protein |
| SMU_1286c | 0.5 | *blt* | multidrug resistance permease |
| SMU_698 | 0.5 | *rpmI* | 50S ribosomal protein L35 |
| SMU_936 | 0.5 | | amino acid ABC transporter, ATP-binding protein |
| SMU_865 | 0.5 | *rpsP* | 30S ribosomal protein S16 |
| SMU_934 | 0.5 | | amino acid ABC transporter, permease protein |
| SMU_961 | 0.5 | | macrophage infectivity potentiator-related protein |
| SMU_1927 | 0.5 | *psaA* | PsaA protein/ ABC transporter, ATP-binding protein |
| SMU_1607 | 0.5 | *rnr* | exoribonuclease R |
| SMU_962 | 0.5 | *mmgC* | acyl-CoA dehydrogenase |
| SMU_935 | 0.5 | | amino acid ABC transporter, permease protein |
| SMU_866 | 0.5 | | conserved hypothetical protein |
| SMU_531 | 0.5 | *pheA* | chorismate mutase; possible prephenate dehydrogenase |
| SMU_614 | 0.4 | | hypothetical protein |
| SMU_364 | 0.4 | *glnA* | glutamine synthetase type 1 |
| SMU_1056 | 0.4 | *radC* | DNA repair protein RadC |
| SMU_1915 | 0.4 | *comC* | S. mutans specific competence stimulating peptide, precursor |
| SMU_365 | 0.4 | *gltA* | glutamate synthase, large subunit |
| SMU_1007 | 0.4 | *ysaB* | ABC transporter permease |
| SMU_613 | 0.4 | | hypothetical protein |
| SMU_1006 | 0.4 | *ysaC* | ABC transporter, ATP-binding protein |
| SMU_1502c | 0.4 | | conserved hypothetical protein |
| SMU_611 | 0.4 | *deaD* | ATP-dependent RNA helicase/DEAD family |
| SMU_396 | 0.3 | *glpF* | glycerol uptake facilitator protein |
| SMU_1175 | 0.3 | *dagA* | sodium:alanine (or glycine) symporter |

**Table S7**. Altered gene expression common to both wild-type and *lrgAB* mutant during heat stress growth (≥ 2-fold change, $P < 0.005$)

| Gene ID | Fold-change (WTh/WT) | Fold-change (ABh/AB) | Gene | Function |
|---|---|---|---|---|
| SMU_263 | 38.7 | 22.1 | *aguD* | Agmatine:putrescine antiporter (Putative amino acid antiporter) |
| SMU_265 | 36.6 | 27.5 | *arcC* | Carbamate kinase |
| SMU_264 | 32.9 | 23.7 | *aguA* | Putative agmatine deiminase (EC 3.5.3.12) (Agmatine iminohydrolase) |
| SMU_262 | 29.4 | 15.5 | *ptcA* | Putrescine carbamoyltransferase (PTC) (PTCase) (EC 2.1.3.6) (Putrescine transcarbamoylase) (Putrescine transcarbamylase) |
| SMU_115 | 15.2 | 9.4 | *fruD* | Constitutive fructose permease (Putative PTS system, fructose-specific IIA component) |
| SMU_116 | 13.5 | 11.5 | *lacD2* | Tagatose 1,6-diphosphate aldolase 2 (EC 4.1.2.40) (D-tagatose-1,6-bisphosphate aldolase 2) (Tagatose-bisphosphate aldolase 2) |
| SMU_114 | 12.8 | 9.7 | *fruC* | Constitutive fructose permease (Putative PTS system, fructose-specific IIBC component) |
| SMU_104 | 11.3 | 5.9 | | Putative alpha-glucosidase glycosyl hydrolase |
| SMU_103 | 10.5 | 5.3 | | Putative PTS system, IIA component |
| SMU_102 | 9.7 | 4.4 | | Putative PTS system, IID component |
| SMU_148 | 8.9 | 2.3 | *adhE* | Aldehyde-alcohol dehydrogenase |
| SMU_105 | 8.2 | 4.8 | | Putative transcriptional regulator repressor of sugar transport operon |
| SMU_113 | 7.8 | 6.4 | *fruP* | Tagatose-6-phosphate kinase (EC 2.7.1.144) |
| SMU_101 | 7 | 2.6 | | Putative sorbose PTS system, IIC component |
| SMU_1068c | 7 | 12 | | Putative ABC transporter, ATP-binding protein |
| SMU_1069c | 7 | 8.8 | | Uncharacterized protein |
| SMU_1067c | 6.8 | 13.8 | | Putative ABC transporter, permease protein |
| SMU_1070c | 5.5 | 5.3 | | Uncharacterized protein |
| SMU_149 | 5.3 | 11.9 | | Putative transposase |
| SMU_1396 | 5.1 | 5 | *gbpC* | Glucan-binding protein C, GbpC |
| SMU_180 | 4.5 | 3.1 | | Putative oxidoreductase possible fumarate reductase (EC 1.3.99.1) |
| SMU_883 | 4.3 | 9.6 | *dexB* | Glucan 1,6-alpha-glucosidase (EC 3.2.1.70) (Dextran glucosidase) (Exo-1,6-alpha-glucosidase) (Glucodextranase) |
| SMU_882 | 4.2 | 8.7 | *msmK* | Multiple sugar-binding transport ATP-binding protein MsmK |
| SMU_1117 | 4.1 | 4 | *naoX* | NADH oxidase (H2O-forming) |
| SMU_881 | 4.1 | 8.4 | *gtfA* | Sucrose phosphorylase (EC 2.4.1.7) (Glucosyltransferase-A) (GTF-A) (Sucrose glucosyltransferase) |
| SMU_1421 | 4 | 4.7 | *pdhC* | Putative dihydrolipoamide acetyltransferase, E2 component (EC 2.3.1.12) |
| SMU_879 | 3.9 | 7.4 | *msmF* | Multiple sugar-binding transport system permease protein MsmF |
| SMU_878 | 3.9 | 6.5 | *msmE* | Multiple sugar-binding protein |
| SMU_880 | 3.8 | 7.7 | *msmG* | Multiple sugar-binding transport system permease protein MsmG |
| SMU_1116c | 3.6 | 4.6 | | Uncharacterized protein |
| SMU_877 | 3.6 | 4.5 | *aga* | Alpha-galactosidase (EC 3.2.1.22) (Melibiase) |

| | | | | |
|---|---|---|---|---|
| SMU_1395c | 3.6 | 2.3 | | Uncharacterized protein |
| SMU_1411 | 3.5 | 8.8 | | Uncharacterized protein |
| SMU_1422 | 3.4 | 3.7 | *pdhB* | Putative pyruvate dehydrogenase E1 component beta subunit (EC 1.2.4.1) |
| SMU_1399 | 3.4 | 2.7 | | Uncharacterized protein |
| SMU_1536 | 3.3 | 5.5 | *glgA* | Glycogen synthase (EC 2.4.1.21) (Starch [bacterial glycogen] synthase) |
| SMU_1423 | 3.2 | 2.9 | *pdhA* | Putative pyruvate dehydrogenase, TPP-dependent E1 component alpha-subunit (EC 1.2.4.1) |
| SMU_1410 | 3.2 | 5.8 | | Putative reductase |
| SMU_1900 | 3.1 | 3 | | Uncharacterized protein |
| SMU_609 | 3 | 6.1 | | Putative 40K cell wall protein |
| SMU_1535 | 3 | 5.03 | *phsG* | Glycogen phosphorylase (EC 2.4.1.1) |
| SMU_1537 | 3 | 5.3 | *glgD* | Putative glycogen biosynthesis protein GlgD |
| SMU_886 | 3 | 3 | *galK* | Galactokinase (EC 2.7.1.6) (Galactose kinase) |
| SMU_1243 | 3 | 2.6 | | Putative low temperature requirement A protein |
| SMU_1004 | 2.9 | 2.6 | *gtfB* | Glucosyltransferase-I (GTF-I) (EC 2.4.1.5) (Dextransucrase) (Sucrose 6-glucosyltransferase) |
| SMU_1599 | 2.8 | 2.3 | *celR* | Putative transcriptional regulator possible antiterminator |
| SMU_1538 | 2.7 | 4.9 | *glgC* | Glucose-1-phosphate adenylyltransferase (EC 2.7.7.27) (ADP-glucose pyrophosphorylase) (ADPGlc PPase) (ADP-glucose synthase) |
| SMU_940c | 2.7 | 2.3 | | Putative hemolysin III |
| SMU_576 | 2.7 | 2.2 | *lytT* | Sensory transduction protein LytT |
| SMU_1844 | 2.7 | 4.6 | *scrR* | Sucrose operon repressor (Scr operon regulatory protein) |
| SMU_984 | 2.7 | 5.8 | | Uncharacterized protein |
| SMU_1596 | 2.7 | 2.7 | *ptcC* | Permease IIC component |
| SMU_1036 | 2.6 | 2.9 | | Uncharacterized protein |
| SMU_985 | 2.5 | 8.8 | *bglA* | Putative beta-glucosidase (EC 3.2.1.86) |
| SMU_616 | 2.4 | 3.7 | | Uncharacterized protein |
| SMU_1898 | 2.4 | 2.7 | | Putative ABC transporter, ATP-binding and permease protein |
| SMU_618 | 2.3 | 3.9 | | Uncharacterized protein |
| SMU_1035 | 2.3 | 3.3 | *glrA* | Putative ABC transporter, ATP-binding protein |
| SMU_1217c | 2.2 | 2.6 | | Putative ABC transporter, amino acid binding protein |
| SMU_1168 | 2.2 | 2.5 | | Putative transcriptional regulator |
| SMU_591c | 2.2 | 3.6 | | Uncharacterized protein |
| SMU_1843 | 2.1 | 2.4 | *scrB* | Sucrose-6-phosphate hydrolase (Sucrase) (EC 3.2.1.26) (Invertase) |
| SMU_1603 | 2.1 | 2.7 | *lguL* | Putative lactoylglutathione lyase (EC 4.4.1.5) |
| SMU_765 | 2.1 | 2.7 | | NADH oxidase/alkyl hydroperoxidase reductase peroxide-forming |
| SMU_1601 | 2.1 | 3.3 | *bgl* | Putative phospho-beta-glucosidase (EC 3.2.1.86) |
| SMU_1571 | 2.1 | 5.3 | | Putative ABC transporter, ATP-binding protein, MsmK-like protein |
| SMU_503c | 2.1 | 3.5 | | Uncharacterized protein |
| SMU_1570 | 2.1 | 4.9 | *malG* | Putative maltose/maltodextrin ABC transporter, MalG permease |
| SMU_592c | 2.1 | 2.9 | | Uncharacterized protein |

| | | | | |
|---|---|---|---|---|
| SMU_1400c | 2 | 2.3 | | Uncharacterized protein |
| SMU_1569 | 2 | 4.4 | *malF* | Putative maltose/maltodextrin ABC transporter, permease protein MalF |
| SMU_1071c | 2 | 2.3 | | Uncharacterized protein |
| SMU_1809 | 2 | 2 | *scnG* | Putative bacteroiocin operon protein ScnG-like protein |
| SMU_422 | 0.5 | 0.31 | *rbfA* | Ribosome-binding factor A |
| SMU_611 | 0.49 | 0.34 | *cshA* | DEAD-box ATP-dependent RNA helicase CshA (EC 3.6.4.13) |
| SMU_1745c | 0.49 | 0.41 | | Putative transcriptional regulator |
| SMU_922 | 0.49 | 0.41 | | Putative ABC transporter, ATP-binding protein |
| SMU_871 | 0.49 | 0.26 | *pfkB* | Tagatose-6-phosphate kinase (EC 2.7.1.144) |
| SMU_1419 | 0.49 | 0.46 | | Putative transcriptional regulator |
| SMU_766 | 0.49 | 0.42 | | Uncharacterized protein |
| SMU_836 | 0.47 | 0.46 | | Uncharacterized protein |
| SMU_2109 | 0.46 | 0.32 | | Putative MDR permease possible multidrug efflux pump |
| SMU_1408c | 0.46 | 0.47 | | Putative transcriptional regulator |
| SMU_934 | 0.45 | 0.22 | | Putative amino acid ABC transporter, permease protein |
| SMU_913 | 0.44 | 0.32 | | Glutamate dehydrogenase |
| SMU_932 | 0.44 | 0.21 | | Uncharacterized protein |
| SMU_63c | 0.43 | 0.19 | | Uncharacterized protein |
| SMU_862 | 0.43 | 0.45 | | Uncharacterized protein |
| SMU_1194 | 0.43 | 0.43 | | Putative ABC transporter, ATP-binding protein |
| SMU_961 | 0.43 | 0.22 | | Alkyl hydroperoxide reductase AhpD (EC 1.11.1.15) |
| SMU_20 | 0.42 | 0.45 | *mreC* | Cell shape-determining protein MreC (Cell shape protein MreC) |
| SMU_936 | 0.42 | 0.23 | | Putative amino acid ABC transporter, ATP-binding protein |
| SMU_935 | 0.42 | 0.23 | | Putative amino acid ABC transporter, permease protein |
| SMU_673 | 0.41 | 0.25 | | Uncharacterized protein |
| SMU_1338c | 0.41 | 0.4 | | Putative permease possible multidrug-efflux transporter |
| SMU_498 | 0.41 | 0.46 | *comF* | Putative late competence protein |
| SMU_1193 | 0.4 | 0.4 | | Putative transcriptional regulator |
| SMU_1985 | 0.39 | 0.48 | *comYB* | Putative ABC transporter ComYB probably part of the DNA transport machinery |
| SMU_625 | 0.39 | 0.45 | *comEAS* | Putative competence protein |
| SMU_1981c | 0.39 | 0.46 | | Uncharacterized protein |
| SMU_1983 | 0.39 | 0.47 | *comYD* | Putative competence protein ComYD |
| SMU_933 | 0.39 | 0.23 | | Putative amino acid ABC transporter, periplasmic amino acid-binding protein |
| SMU_1827 | 0.39 | 0.41 | | Putative biotin biosynthesis protein |
| SMU_1982c | 0.37 | 0.43 | | Uncharacterized protein |
| SMU_626 | 0.36 | 0.42 | | Putative competence protein |
| SMU_1379 | 0.36 | 0.39 | | Uncharacterized protein |
| SMU_921 | 0.35 | 0.3 | | Putative transcriptional regulator |
| SMU_1286c | 0.34 | 0.29 | | Putative permease multidrug efflux protein |
| SMU_1975c | 0.33 | 0.31 | | Uncharacterized protein |
| SMU_1954 | 0.33 | 0.41 | *groEL* | 60 kDa chaperonin (GroEL protein) (Protein Cpn60) |
| SMU_962 | 0.33 | 0.16 | | Putative dehydrogenase |

| | | | | |
|---|---|---|---|---|
| SMU_1284c | 0.33 | 0.3 | | Uncharacterized protein |
| SMU_81 | 0.32 | 0.47 | *grpE* | Protein GrpE (HSP-70 cofactor) |
| SMU_1175 | 0.31 | 0.19 | | Putative sodium/amino acid (Alanine) symporter |
| SMU_1976c | 0.31 | 0.25 | | Uncharacterized protein |
| SMU_80 | 0.3 | 0.4 | *hrcA* | Heat-inducible transcription repressor HrcA |
| SMU_672 | 0.3 | 0.2 | *icd* | Isocitrate dehydrogenase [NADP] (IDH) (EC 1.1.1.42) (IDP) (NADP(+)-specific ICDH) (Oxalosuccinate decarboxylase) |
| SMU_872 | 0.3 | 0.29 | *fruI* | Inducible fructose permease (Putative PTS system, fructose-specific enzyme IIABC component) |
| SMU_1977c | 0.3 | 0.24 | | Putative transcriptional regulator |
| SMU_1997 | 0.3 | 0.4 | *comX1* | Putative ComX1, transcriptional regulator of competence-specific genes |
| SMU_2133c | 0.29 | 0.15 | | Putative membrane protein |
| SMU_1955 | 0.29 | 0.34 | *groES* | 10 kDa chaperonin (GroES protein) (Protein Cpn10) |
| SMU_671 | 0.27 | 0.19 | *citZ* | Citrate synthase (EC 2.3.3.16) |
| SMU_1584c | 0.27 | 0.15 | | Putative 67 kDa myosin-crossreactive streptococcal antigen-like protein |
| SMU_1658 | 0.26 | 0.29 | *nrgA* | Ammonium transporter |
| SMU_1657c | 0.26 | 0.19 | | Putative nitrogen regulatory protein PII |
| SMU_670 | 0.23 | 0.27 | *citB* | Aconitate hydratase A (ACN) (Aconitase) (EC 4.2.1.3) ((2R,3S)-2-methylisocitrate dehydratase) ((2S,3R)-3-hydroxybutane-1,2,3-tricarboxylate dehydratase) (Iron-responsive protein-like) (IRP-like) (Probable 2-methyl-cis-aconitate hydratase) (EC 4.2.1.99) (RNA-binding protein) |
| SMU_1348c | 0.21 | 0.37 | | Putative ABC transporter ATP-binding protein (Putative ABC transporter, ATP-binding protein) |
| SMU_1366c | 0.2 | 0.37 | | Putative ABC transporter ATP-binding protein (Putative ABC transporter, ATP-binding protein) |
| SMU_1345c | 0.12 | 0.48 | | Putative peptide synthetase |
| SMU_1344c | 0.1 | 0.47 | | Putative malonyl-CoA acyl-carrier-protein transacylase (EC 2.3.1.39) |
| SMU_1343c | 0.09 | 0.47 | | Putative polyketide synthase |
| SMU_1342 | 0.07 | 0.32 | *bacA1* | Putative bacitracin synthetase 1 BacA |
| SMU_1341c | 0.06 | 0.23 | | Putative gramicidin S synthetase |
| SMU_1339 | 0.05 | 0.21 | *bacD* | Putative bacitracin synthetase |
| SMU_1340 | 0.05 | 0.21 | *bacA2* | Putative surfactin synthetase |

**Table S8.** Altered gene expression unique to wild-type during heat stress growth (≥ 2-fold change, $P < 0.005$)

| Gene ID | Fold-change (WTh/WT) | Gene | Function |
|---|---|---|---|
| SMU_79 | 7.9 | *fruB* | Inducible Fructanase (EC 3.2.1.80), FruB |
| SMU_78 | 6.5 | *fruA* | Sucrose-6-phosphate hydrolase (EC 3.2.1.26) |
| SMU_575c | 4.0 | *lrgA* | Antiholin-like protein LrgA |
| SMU_1956c | 3.8 | | PTS system, fructose- and mannose-inducible putative EII component |
| SMU_1957 | 3.7 | *levG* | PTS system, fructose- and mannose-inducible IID component (EC 2.7.1.69) |
| SMU_1958c | 3.6 | *levF* | PTS system, fructose- and mannose-inducible IIC component (EC 2.7.1.69) |
| SMU_2127 | 3.4 | *gabD* | Succinate-semialdehyde dehydrogenase [NAD] (EC 1.2.1.24); Succinate-semialdehyde dehydrogenase [NADP+] (EC 1.2.1.16) |
| SMU_1960c | 3.1 | *levE* | PTS system, fructose- and mannose-inducible IIB component (EC 2.7.1.69) |
| SMU_1961c | 3.1 | *levD* | PTS system, fructose- and mannose-inducible IIA component (EC 2.7.1.69) |
| SMU_1896c | 3.0 | | hypothetical protein |
| SMU_100 | 3.0 | *sorD* | Phosphotransferase system, mannose/fructose/N-acetylgalactosamine-specific component IIB |
| SMU_574c | 2.9 | *lrgB* | LrgA-associated membrane protein LrgB |
| SMU_402 | 2.9 | *pfl* | Pyruvate formate-lyase (EC 2.3.1.54) |
| SMU_1895c | 2.8 | | FIG01120352: hypothetical protein |
| SMU_1425 | 2.7 | *clpB* | ClpB protein |
| SMU_179 | 2.6 | | Fumarate reductase, flavoprotein subunit precursor (EC 1.3.99.1) |
| SMU_982 | 2.4 | *bglB* | FIG01120188: hypothetical protein |
| SMU_2028 | 2.4 | *ftf* | Fructosyltransfearse Ftf |
| SMU_270 | 2.4 | *rmpC* | Ascorbate-specific PTS system, EIIC component |
| SMU_577 | 2.3 | *lytS* | Autolysis histidine kinase LytS |
| SMU_1259 | 2.3 | | type II restriction endonuclease, putative |
| SMU_1600 | 2.2 | *celB* | PTS system, cellobiose-specific IIB component (EC 2.7.1.69) |
| SMU_798c | 2.2 | | FIG01117437: hypothetical protein |
| SMU_1598 | 2.2 | *celC* | PTS system, cellobiose-specific IIA component (EC 2.7.1.69) |
| SMU_1597c | 2.2 | | FIG01114705: hypothetical protein |
| SMU_1398 | 2.2 | *irvR* | hypothetical protein |
| SMU_1424 | 2.1 | *adhD* | Dihydrolipoamide dehydrogenase of acetoin dehydrogenase (EC 1.8.1.4) |
| SMU_876 | 2.2 | *msmR* | MSM (multiple sugar metabolism) operon regulatory protein |
| SMU_133c | 2.1 | | FIG01119245: hypothetical protein |
| SMU_89c | 2.1 | | Formate-nitrate transporter |
| SMU_1810 | 2.1 | *scnE* | FIG01116767: hypothetical protein |
| SMU_1282 | 2.1 | *psaR* | Transcriptional regulator, TetR family |
| SMU_438c | 2.1 | | Activator of (R)-2-hydroxyglutaryl-CoA dehydratase |
| SMU_1811 | 2.1 | *scnF* | ABC-type multidrug transport system, ATPase component |

| | | | |
|---|---|---|---|
| SMU_1075 | 2.0 | *dfp* | Phosphopantothenoylcysteine decarboxylase (EC 4.1.1.36) |
| SMU_1908c | 0.5 | | hypothetical protein |
| SMU_602 | 0.5 | | Probable Na+ dependent transporter possibly for ileal bile acids |
| SMU_1086 | 0.5 | *tdk* | Thymidine kinase (EC 2.7.1.21) |
| SMU_109 | 0.5 | | FIG01119058: hypothetical protein |
| SMU_1195 | 0.5 | | ABC transporter, ATP-binding protein |
| SMU_866 | 0.5 | | KH domain RNA binding protein YlqC |
| SMU_863 | 0.5 | | ABC transporter ATP-binding protein |
| SMU_1420 | 0.5 | *qr1* | hypothetical protein |
| SMU_499 | 0.5 | *comFC* | ComF operon protein C |
| SMU_1903c | 0.4 | | |
| SMU_1987 | 0.4 | *comGA* | Late competence protein ComGA, access of DNA to ComEA |
| SMU_1001 | 0.4 | *dprA* | Rossmann fold nucleotide-binding protein Smf possibly involved in DNA uptake |
| SMU_1984 | 0.4 | *comGC* | Late competence protein ComGC, access of DNA to ComEA, FIG007487 |
| SMU_1980c | 0.4 | | Late competence protein ComGG, FIG068335 |
| SMU_83 | 0.4 | *dnaJ* | Chaperone protein DnaJ |
| SMU_1706 | 0.3 | | FIG015389: hypothetical membrane associated protein |
| SMU_82 | 0.3 | *dnaK* | Chaperone protein DnaK |
| SMU_1185 | 0.3 | *mtlA* | PTS system, mannitol-specific IIB component (EC 2.7.1.69) / PTS system, mannitol-specific IIC component (EC 2.7.1.69) |
| SMU_1705 | 0.3 | | FIG04612: Integral membrane protein (putative) |
| SMU_1704 | 0.2 | | FIG014387: Transcriptional regulator, PadR family |
| SMU_1365c | 0.2 | | ABC transporter permease protein |
| SMU_1347c | 0.2 | | ABC transporter permease protein |
| SMU_1346 | 0.1 | *bacT* | Thioesterase |

**Table S9.** Altered gene expression unique to *lrgAB* mutant during heat stress growth (≥ 2-fold change, *P*< 0.005)

| Gene ID | Fold-change (ABh/AB) | Gene | Function |
|---|---|---|---|
| SMU_1023 | 4.1 | *pycB* | Oxaloacetate decarboxylase alpha chain (EC 4.1.1.3) |
| SMU_1021 | 3.8 | *citF* | Citrate lyase alpha chain (EC 4.1.3.6) |
| SMU_1564 | 3.8 | *glg* | Maltodextrin phosphorylase (EC 2.4.1.1) |
| SMU_1022 | 3.7 | *citX2* | Apo-citrate lyase phosphoribosyl-dephospho-CoA transferase (EC 2.7.7.61) |
| SMU_1013c | 3.4 | *yxiQ* | Mg2+/citrate complex transporter |
| SMU_1568 | 3.3 | *malE* | Maltose/maltodextrin ABC transporter, substrate binding periplasmic protein MalE |
| SMU_1020 | 3.1 | *citE* | Citrate lyase beta chain (EC 4.1.3.6) |
| SMU_1037c | 3.0 | *phoR* | putative histidine kinase |
| SMU_1404c | 3.0 | | CRISPR-associated protein Cas1 |
| SMU_1038c | 2.9 | *vicR* | Two-component system response regulator |
| SMU_375 | 2.9 | | FIG00630265: hypothetical protein |
| SMU_1539 | 2.9 | *glgB* | 1,4-alpha-glucan (glycogen) branching enzyme, GH-13-type (EC 2.4.1.18) |
| SMU_1403c | 2.8 | | CRISPR-associated protein Cas2 |
| SMU_1565 | 2.8 | *malM* | 4-alpha-glucanotransferase (amylomaltase) (EC 2.4.1.25) |
| SMU_376 | 2.7 | | Adenosylmethionine-8-amino-7-oxononanoate aminotransferase (EC 2.6.1.62) |
| SMU_1405c | 2.7 | | CRISPR-associated protein, Csn1 family |
| SMU_2027 | 2.7 | | Pleiotropic regulator of exopolysaccharide synthesis, competence and biofilm formation Ftr, XRE family |
| SMU_1016 | 2.6 | *accB* | Biotin carboxyl carrier protein of oxaloacetate decarboxylase; Biotin carboxyl carrier protein |
| SMU_1402c | 2.6 | | CRISPR-associated protein, Csn2 family |
| SMU_1218 | 2.6 | *gatA* | 6-aminohexanoate-cyclic-dimer hydrolase (EC 3.5.2.12) |
| SMU_620 | 2.6 | | |
| SMU_860 | 2.6 | *carB* | Carbamoyl-phosphate synthase large chain (EC 6.3.5.5) |
| SMU_590c | 2.5 | | Mobile element protein |
| SMU_1010 | 2.5 | *citC* | [Citrate [pro-3S]-lyase] ligase (EC 6.2.1.22) |
| SMU_1017 | 2.5 | *oadB* | Oxaloacetate decarboxylase beta chain (EC 4.1.1.3) |
| SMU_140 | 2.5 | *gshR* | Glutathione reductase (EC 1.8.1.7) |
| SMU_857 | 2.5 | *pyrP* | Uracil permease |
| SMU_400 | 2.5 | | Beta-lactamase, class C |
| SMU_205c | 2.5 | | hypothetical protein |
| SMU_1019 | 2.5 | *citD* | Citrate lyase gamma chain, acyl carrier protein (EC 4.1.3.6) |
| SMU_764 | 2.5 | *ahpC* | Alkyl hydroperoxide reductase protein C (EC 1.6.4.-) |

| | | | |
|---|---|---|---|
| SMU_374 | 2.5 | | Short chain dehydrogenase |
| SMU_200c | 2.4 | | hypothetical protein |
| SMU_238c | 2.4 | | Methionine ABC transporter ATP-binding protein |
| SMU_373 | 2.4 | | FIG01230468: hypothetical protein |
| SMU_202c | 2.4 | | FIG01117874: hypothetical protein |
| SMU_859 | 2.4 | *carA* | Carbamoyl-phosphate synthase small chain (EC 6.3.5.5) |
| SMU_911c | 2.4 | | FIG01119515: hypothetical protein |
| SMU_2111c | 2.4 | *orf1* | FIG01115672: hypothetical protein |
| SMU_1039c | 2.3 | *kdt* | UDP-galactose:(galactosyl) LPS alpha1,2-galactosyltransferase WaaW (EC 2.4.1.-) |
| SMU_1561 | 2.3 | *trkB* | Trk system potassium uptake protein TrkA |
| SMU_204c | 2.3 | | hypothetical protein |
| SMU_1488c | 2.3 | | FIG01114080: hypothetical protein |
| SMU_199c | 2.3 | | hypothetical protein |
| SMU_1562 | 2.3 | *trkA* | Trk system potassium uptake protein TrkA |
| SMU_953c | 2.3 | | Predicted transcriptional regulator of pyridoxine metabolism |
| SMU_1490 | 2.3 | *lacG* | 6-phospho-beta-galactosidase (EC 3.2.1.85) |
| SMU_25 | 2.3 | *recO* | DNA recombination and repair protein RecO |
| SMU_185 | 2.3 | | |
| SMU_193c | 2.3 | | hypothetical protein |
| SMU_1011 | 2.3 | *citG* | 2-(5''-triphosphoribosyl)-3'-dephosphocoenzyme-A synthase (EC 2.7.8.25) |
| SMU_858 | 2.2 | *pyrB* | Aspartate carbamoyltransferase (EC 2.1.3.2) |
| SMU_1988c | 2.2 | | DNA binding protein, FIG046916 |
| SMU_1018 | 2.2 | | FIG01114213: hypothetical protein |
| SMU_1602 | 2.2 | *frp* | Oxygen-insensitive NAD(P)H nitroreductase (EC 1.-.-.-) / Dihydropteridine reductase (EC 1.5.1.34) |
| SMU_138 | 2.2 | *mleP* | Malate permease |
| SMU_1219c | 2.2 | | FIG01120054: hypothetical protein |
| SMU_1409c | 2.2 | *bglC* | Transcriptional regulator, AraC family |
| SMU_196c | 2.1 | | putative transfer protein |
| SMU_646 | 2.1 | *gph* | hydrolase, haloacid dehalogenase-like family |
| SMU_1388 | 2.1 | *deaD* | ATP-dependent RNA helicase YfmL |
| SMU_1012c | 2.1 | *cpsY* | Transcriptional regulator, GntR family |
| SMU_1014 | 2.1 | | FIG01114846: hypothetical protein |
| SMU_1389 | 2.1 | *pckA* | Phosphoenolpyruvate carboxykinase [ATP] (EC 4.1.1.49) |
| SMU_887 | 2.1 | *galT* | Galactose-1-phosphate uridylyltransferase (EC 2.7.7.10) |
| SMU_1833 | 2.1 | *recG* | ATP-dependent DNA helicase RecG (EC 3.6.1.-) |
| SMU_141 | 2.1 | | integral membrane protein |
| SMU_1862 | 2.1 | | |
| SMU_210c | 2.1 | | hypothetical protein |

| | | | |
|---|---|---|---|
| SMU_637c | 2.1 | | hypothetical protein |
| SMU_2047 | 2.1 | *ptsG* | PTS system, enzyme II, A component |
| SMU_1753c | 2.1 | | CRISPR-associated protein Cas2 |
| SMU_283 | 2.0 | | FIG01116676: hypothetical protein |
| SMU_139 | 2.0 | *oxdC* | Oxalate decarboxylase (EC 4.1.1.2) |
| SMU_1574c | 2.0 | | conserved hypothetical protein |
| SMU_197c | 2.0 | | hypothetical protein |
| SMU_1005 | 2.0 | *gtfC* | Glucosyltransferase GtfG |
| SMU_1040c | 2.0 | *ydfG* | Short chain dehydrogenase |
| SMU_954 | 0.5 | *pdxK* | Pyridoxal kinase (EC 2.7.1.35) |
| SMU_2002 | 0.5 | *rs11* | SSU ribosomal protein S11p (S14e) |
| SMU_227c | 0.5 | | hypothetical protein |
| SMU_1627 | 0.5 | *rplK* | LSU ribosomal protein L11p (L12e) |
| SMU_2003 | 0.5 | *rs13* | SSU ribosomal protein S13p (S18e) |
| SMU_154 | 0.5 | *rpsO* | SSU ribosomal protein S15p (S13e) |
| SMU_1626 | 0.5 | *rplA* | LSU ribosomal protein L1p (L10Ae) |
| SMU_2033c | 0.5 | | FIG01116025: hypothetical protein |
| SMU_2031 | 0.5 | *tfs* | Translation elongation factor Ts |
| SMU_849 | 0.5 | *rpmA* | LSU ribosomal protein L27p |
| SMU_421 | 0.5 | *infB* | Translation initiation factor 2 |
| SMU_419 | 0.5 | | COG2740: Predicted nucleic-acid-binding protein implicated in transcription termination |
| SMU_1063 | 0.5 | *atmD* | Glycine betaine ABC transport system, ATP-binding protein OpuAA (EC 3.6.3.32) |
| SMU_2147c | 0.5 | | Aggregation promoting factor |
| SMU_2001 | 0.5 | *rpoA* | DNA-directed RNA polymerase alpha subunit (EC 2.7.7.6) |
| SMU_1879 | 0.5 | *ptnD* | PTS system, mannose-specific IID component (EC 2.7.1.69) |
| SMU_2003a | 0.5 | *rl36* | LSU ribosomal protein L36p |
| SMU_2000 | 0.5 | *rl17* | LSU ribosomal protein L17p |
| SMU_2004 | 0.5 | *if1* | Translation initiation factor 1 |
| SMU_1859 | 0.5 | *ssb* | Single-stranded DNA-binding protein |
| SMU_420 | 0.5 | | ribosomal protein L7Ae family protein |
| SMU_85 | 0.5 | *thiD* | Novel pyridoxal kinase, thiD family (EC 2.7.1.35) |
| SMU_846 | 0.5 | *rplU* | LSU ribosomal protein L21p |
| SMU_1858 | 0.5 | *rs18* | SSU ribosomal protein S18p @ SSU ribosomal protein S18p, zinc-independent |
| SMU_1937 | 0.5 | *cnhA* | Aliphatic amidase amiE (EC 3.5.1.4) |
| SMU_1610 | 0.5 | *rpmG* | LSU ribosomal protein L33p @ LSU ribosomal protein L33p, zinc-dependent |
| SMU_16 | 0.5 | | Cationic amino acid transporter - APC Superfamily |
| SMU_1878 | 0.5 | *ptnC* | PTS system, mannose-specific IIC component (EC 2.7.1.69) |

| | | | |
|---|---|---|---|
| SMU_1938c | 0.5 | *atmE* | Methionine ABC transporter permease protein |
| SMU_699 | 0.4 | *rplT* | LSU ribosomal protein L20p |
| SMU_848 | 0.4 | | FIG139598: Potential ribosomal protein |
| SMU_84 | 0.4 | *truA* | tRNA pseudouridine synthase A (EC 4.2.1.70) |
| SMU_910 | 0.4 | *gtfD* | Glucosyltransferase GtfG |
| SMU_600c | 0.4 | | Substrate-specific component FolT of folate ECF transporter |
| SMU_108 | 0.4 | | |
| SMU_698 | 0.4 | *rpmI* | LSU ribosomal protein L35p |
| SMU_2037 | 0.4 | *treC* | Trehalose-6-phosphate hydrolase (EC 3.2.1.93) |
| SMU_2020 | 0.4 | *rl16* | LSU ribosomal protein L16p (L10e) |
| SMU_2167 | 0.4 | *rplB* | |
| SMU_128 | 0.4 | *adhB* | Acetoin dehydrogenase E1 component beta-subunit (EC 1.2.4.-) |
| SMU_2009 | 0.4 | *rs5* | SSU ribosomal protein S5p (S2e) |
| SMU_2032 | 0.4 | *rs2* | SSU ribosomal protein S2p (SAe) |
| SMU_2162c | 0.4 | | LSU m3Psi1915 methyltransferase RlmH |
| SMU_364 | 0.4 | *glnA* | Glutamine synthetase type I (EC 6.3.1.2) |
| SMU_120 | 0.4 | *rl28* | LSU ribosomal protein L28p |
| SMU_365 | 0.4 | *gltA* | Glutamate synthase [NADPH] large chain (EC 1.4.1.13) |
| SMU_1940c | 0.4 | *atmC* | Catalyzes the cleavage of p-aminobenzoyl-glutamate to p-aminobenzoate and glutamate, subunit A |
| SMU_1939c | 0.4 | *atmD* | Methionine ABC transporter ATP-binding protein |
| SMU_2007 | 0.4 | *rl15* | LSU ribosomal protein L15p (L27Ae) |
| SMU_459 | 0.4 | | Cysteine ABC transporter, substrate-binding protein |
| SMU_460 | 0.4 | | Cysteine ABC transporter, permease protein |
| SMU_2021 | 0.4 | *rs3* | SSU ribosomal protein S3p (S3e) |
| SMU_1288 | 0.4 | *rplS* | LSU ribosomal protein L19p |
| SMU_2017 | 0.4 | *rl14* | LSU ribosomal protein L14p (L23e) |
| SMU_2014 | 0.4 | *rs14* | SSU ribosomal protein S14p (S29e) |
| SMU_2016 | 0.4 | *rl24* | LSU ribosomal protein L24p (L26e) |
| SMU_169 | 0.4 | *rplM* | LSU ribosomal protein L13p (L13Ae) |
| SMU_2023c | 0.4 | *rps19* | SSU ribosomal protein S19p (S15e) |
| SMU_2010 | 0.4 | *rl18* | LSU ribosomal protein L18p (L5e) |
| SMU_2022 | 0.4 | *rl22* | LSU ribosomal protein L22p (L17e) |
| SMU_2026c | 0.4 | *rps1NA* | SSU ribosomal protein S10p (S20e) |
| SMU_1894c | 0.4 | | Mobile element protein |
| SMU_697 | 0.4 | *infC* | Translation initiation factor 3 |
| SMU_2008 | 0.4 | *rl3NA* | LSU ribosomal protein L30p (L7e) |
| SMU_2015 | 0.4 | *rl5* | LSU ribosomal protein L5p (L11e) |
| SMU_1941 | 0.4 | *atmB* | Methionine ABC transporter substrate-binding protein |
| SMU_2024c | 0.4 | *rl4* | LSU ribosomal protein L4p (L1e) |

| | | | |
|---|---|---|---|
| SMU_2006 | 0.4 | *secY* | Preprotein translocase secY subunit (TC 3.A.5.1.1) |
| SMU_2011 | 0.4 | *rl6* | LSU ribosomal protein L6p (L9e) |
| SMU_461 | 0.4 | | ABC-type multidrug transport system, ATPase component |
| SMU_2018 | 0.4 | *rs17* | SSU ribosomal protein S17p (S11e) |
| SMU_2166 | 0.4 | *rplW* | |
| SMU_170 | 0.4 | *rpsI* | SSU ribosomal protein S9p (S16e) |
| SMU_2012 | 0.4 | *rs8* | SSU ribosomal protein S8p (S15Ae) |
| SMU_2025 | 0.4 | *rl3* | LSU ribosomal protein L3p (L3e) |
| SMU_2019 | 0.4 | *rl29* | LSU ribosomal protein L29p (L35e) |
| SMU_566c | 0.4 | | Mobile element protein |
| SMU_818 | 0.4 | *rpsU* | SSU ribosomal protein S21p |
| SMU_2038 | 0.4 | *treB* | PTS system, trehalose-specific IIB component (EC 2.7.1.69) / PTS system, trehalose-specific IIC component (EC 2.7.1.69) / PTS system, trehalose-specific IIA component (EC 2.7.1.69) |
| SMU_1502c | 0.3 | | FIG01118403: hypothetical protein |
| SMU_71 | 0.3 | | Multi antimicrobial extrusion (MATE) family transporter |
| SMU_1390 | 0.3 | | FIG01119820: hypothetical protein |
| SMU_1877 | 0.3 | *manL* | PTS system, mannose-specific IIB component (EC 2.7.1.69) / PTS system, mannose-specific IIA component (EC 2.7.1.69) |
| SMU_1342 | 0.3 | *bacA* | Long-chain-fatty-acid--CoA ligase (EC 6.2.1.3) |
| SMU_496 | 0.3 | *cysK* | Cysteine synthase (EC 2.5.1.47) |
| SMU_958 | 0.3 | | |
| SMU_1545c | 0.3 | | Substrate-specific component QueT (COG4708) of predicted queuosine-regulated ECF transporter |
| SMU_1856c | 0.3 | | Conserved Membrane Protein (Archaea) |
| SMU_870 | 0.3 | *fruR* | Transcriptional repressor of the fructose operon, DeoR family |
| SMU_960 | 0.3 | *rpl* | LSU ribosomal protein L7/L12 (P1/P2) |
| SMU_1595 | 0.3 | *cah* | Carbonic anhydrase (EC 4.2.1.1) |
| SMU_957 | 0.3 | *rplJ* | LSU ribosomal protein L10p (P0) |
| SMU_1927 | 0.2 | *psaA* | ABC transporter ATP-binding protein YvcR |

**Table S10.** Altered gene expression common to both wild-type and *lrgAB* mutant during vancomycin growth (≥ 1.5-fold change, $P < 0.005$)

| Gene ID | Fold-change (WTv/WT) | Fold-change (ABv/AB) | Gene | Function |
|---|---|---|---|---|
| SMU_932 | 6.7 | 4.7 | | Uncharacterized protein |
| SMU_933 | 5.5 | 3.8 | | Putative amino acid ABC transporter, periplasmic amino acid-binding protein |
| SMU_1094 | 4.7 | 13.9 | | Putative ABC transporter, ATP-binding protein |
| SMU_934 | 4.6 | 3.2 | | Putative amino acid ABC transporter, permease protein |
| SMU_935 | 4.2 | 2.6 | | Putative amino acid ABC transporter, permease protein |
| SMU_936 | 3.9 | 2.5 | | Putative amino acid ABC transporter, ATP-binding protein |
| SMU_961 | 3.5 | 2.2 | | Alkyl hydroperoxide reductase AhpD (EC 1.11.1.15) |
| SMU_1093 | 3.5 | 11.6 | | Putative ABC transporter, permease protein |
| SMU_962 | 3.2 | 2 | | Putative dehydrogenase |
| SMU_1421 | 2.3 | 3 | *pdhC* | Dihydrolipoamide acetyltransferase component of pyruvate dehydrogenase complex (EC 2.3.1.-) |
| SMU_930c | 2.3 | 2 | | Putative transcriptional regulator |
| SMU_1422 | 2.2 | 22.7 | *pdhB* | Putative pyruvate dehydrogenase E1 component beta subunit (EC 1.2.4.1) |
| SMU_1599 | 2 | 1.7 | *celR* | Putative transcriptional regulator possible antiterminator |
| SMU_1423 | 2 | 2.5 | *pdhA* | Putative pyruvate dehydrogenase, TPP-dependent E1 component alpha-subunit (EC 1.2.4.1) |
| SMU_1596 | 1.9 | 1.8 | *ptcC* | Permease IIC component |
| SMU_651c | 1.9 | 2.5 | | Putative ABC transporter, substrate-binding protein |
| SMU_1602 | 1.8 | 2.5 | | Putative NAD(P)H-flavin oxidoreductase |
| SMU_653c | 1.8 | 1.9 | | Putative ABC transporter, permease protein |
| SMU_652c | 1.8 | 2.3 | | Putative ABC transporter, ATP-binding protein possible nitrate transport system |
| SMU_1601 | 1.6 | 1.6 | *bgl* | Putative phospho-beta-glucosidase (EC 3.2.1.86) |
| SMU_1493 | 1.6 | 1.6 | *lacD* | Tagatose 1,6-diphosphate aldolase 1 (EC 4.1.2.40) (D-tagatose-1,6-bisphosphate aldolase 1) (Tagatose-bisphosphate aldolase 1) |
| SMU_1494 | 1.6 | 1.6 | *lacC* | Tagatose-6-phosphate kinase (EC 2.7.1.144) (Phosphotagatokinase) |
| SMU_114 | 1.6 | 1.7 | *fruC* | Constitutive fructose permease (Putative PTS system, fructose-specific IIBC component) |
| SMU_1914c | 1.5 | 3 | | Uncharacterized protein |
| SMU_1400c | 1.5 | 1.7 | | Uncharacterized protein |
| SMU_1915 | 0.66 | 0.56 | *comC* | Competence stimulating peptide |
| SMU_1419 | 0.65 | 0.59 | | Putative transcriptional regulator |
| SMU_836 | 0.65 | 0.64 | | Uncharacterized protein |
| SMU_1420 | 0.65 | 0.67 | | Putative oxidoreductase (EC 1.6.5.2) |
| SMU_1395c | 0.62 | 0.59 | | Uncharacterized protein |
| SMU_29 | 0.6 | 0.47 | *purC* | Phosphoribosylaminoimidazole-succinocarboxamide synthase (EC 6.3.2.6) (SAICAR synthetase) |
| SMU_625 | 0.58 | 0.59 | *comEA* | Putative competence protein |
| SMU_1927 | 0.58 | 0.38 | | Putative ABC transporter, ATP-binding protein |
| SMU_626 | 0.53 | 0.56 | | Putative competence protein |

| | | | | |
|---|---|---|---|---|
| SMU_498 | 0.51 | 0.56 | *comF* | Putative late competence protein |
| SMU_1984 | 0.51 | 0.61 | *comYC* | Putative competence protein ComYC |
| SMU_1981c | 0.5 | 0.55 | | Uncharacterized protein |
| SMU_1980c | 0.46 | 0.58 | | Uncharacterized protein |
| SMU_1983 | 0.46 | 0.57 | *comYD* | Putative competence protein ComYD |
| SMU_1982c | 0.46 | 0.54 | | Uncharacterized protein |
| SMU_1987 | 0.45 | 0.59 | *comYA* | Putative ABC transporter, ATP-binding protein ComYA late competence protein |
| SMU_1985 | 0.44 | 0.57 | *comYB* | Putative ABC transporter ComYB probably part of the DNA transport machinery |
| SMU_1001 | 0.43 | 0.59 | *smf* | Putative DNA processing Smf protein |
| SMU_1185 | 0.42 | 0.61 | *mtlA* | PTS system mannitol-specific EIICB component (EIICB-Mtl) (EII-Mtl) [Includes: Mannitol permease IIC component (PTS system mannitol-specific EIIC component); Mannitol-specific phosphotransferase enzyme IIB component (EC 2.7.1.197) (PTS system mannitol-specific EIIB component)] |
| SMU_1584c | 0.27 | 0.17 | | Putative 67 kDa myosin-crossreactive streptococcal antigen-like protein |
| SMU_2133c | 0.26 | 0.17 | | Putative membrane protein |

**Table S11.** Altered gene expression unique to wild-type during vancomycin growth (≥ 1.5-fold change, $P < 0.005$)

| Gene ID | Fold-change (WTv/WT) | Gene | Function |
|---|---|---|---|
| SMU_574c | 2.3 | *lrgB* | Putative membrane protein |
| SMU_496 | 1.8 | *cysK* | Cysteine synthase (EC 2.5.1.47) |
| SMU_184 | 1.8 | *sloC* | Metal ABC transporter substrate-binding lipoprotein |
| SMU_183 | 1.8 | *sloB* | Putative Mn/Zn ABC transporter |
| SMU_1598 | 1.7 | *ptcA* | Putative PTS system, cellobiose-specific IIA component |
| SMU_265 | 1.7 | *arcC* | Carbamate kinase |
| SMU_185 | 1.64 | | Uncharacterized protein |
| SMU_672 | 1.6 | *icd* | Isocitrate dehydrogenase [NADP] (IDH) (EC 1.1.1.42) (IDP) (NADP(+)-specific ICDH) (Oxalosuccinate decarboxylase) |
| SMU_671 | 1.6 | *citZ* | Citrate synthase (EC 2.3.3.16) |
| SMU_670 | 1.6 | *citB* | Aconitate hydratase A (ACN) (Aconitase) |
| SMU_1942c | 1.5 | | Putative amino acid binding protein |
| SMU_186 | 1.5 | *sloR* | Putative metal-dependent transcriptional regulator |
| SMU_1013c | 1.5 | | Putative Mg2+/citrate transporter |
| SMU_1597c | 1.5 | | Uncharacterized protein |
| SMU_1967 | 0.87 | *ssb2* | Single-stranded DNA-binding protein (SSB) |
| SMU_1004 | 0.65 | *gtfB* | Glucosyltransferase-I (GTF-I) (EC 2.4.1.5) (Dextransucrase) (Sucrose 6-glucosyltransferase) |
| SMU_100 | 0.65 | | Putative sorbose PTS system, IIB component |
| SMU_209c | 0.64 | | Uncharacterized protein |
| SMU_200c | 0.63 | | Uncharacterized protein |
| SMU_101 | 0.62 | | Putative sorbose PTS system, IIC component |
| SMU_208c | 0.62 | | Putative transposon protein possible DNA segregation ATPase |
| SMU_196c | 0.62 | | Putative transfer protein |
| SMU_197c | 0.59 | | Uncharacterized protein |
| SMU_1892c | 0.59 | | Uncharacterized protein |
| SMU_198c | 0.58 | | Putative conjugative transposon protein |
| SMU_499 | 0.57 | | Putative late competence protein |
| SMU_210c | 0.57 | | Uncharacterized protein |
| SMU_1072c | 0.56 | | Putative acetyltransferase |
| SMU_179 | 0.55 | | Uncharacterized protein |
| SMU_201c | 0.54 | | Putative transposon protein |
| SMU_193c | 0.53 | | Uncharacterized protein |
| SMU_199c | 0.52 | | Uncharacterized protein |
| SMU_202c | 0.51 | | Uncharacterized protein |
| SMU_205c | 0.49 | | Uncharacterized protein |
| SMU_204c | 0.41 | | Uncharacterized protein |
| SMU_1368 | 0.4 | | Uncharacterized protein |

**Table S12.** Altered gene expression unique to *lrgAB* mutant during vancomycin growth (≥ 1.5-fold change, $P < 0.005$)

| Gene ID | Fold-change (ABv/AB) | Gene | Function |
|---|---|---|---|
| SMU_1906c | 2.8 | | Uncharacterized protein |
| SMU_1909c | 2.4 | | Uncharacterized protein |
| SMU_150 | 2.4 | | Uncharacterized protein |
| SMU_1910c | 2.2 | | Uncharacterized protein |
| SMU_151 | 2.2 | | Uncharacterized protein |
| SMU_739c | 2.2 | | Uncharacterized protein |
| SMU_1913c | 2.1 | | Putative immunity protein, BLpL-like |
| SMU_1603 | 2.1 | *lguL* | Putative lactoylglutathione lyase (EC 4.4.1.5) |
| SMU_1908c | 2.1 | | Uncharacterized protein |
| SMU_1912c | 2 | | Uncharacterized protein |
| SMU_423 | 2 | | Uncharacterized protein |
| SMU_138 | 2 | | Putative malate permease |
| SMU_173 | 1.9 | *mazF* | Putative ppGpp-regulated growth inhibitor |
| SMU_1905c | 1.9 | | Putative bacteriocin secretion protein |
| SMU_137 | 1.9 | *mleS* | Malolactic enzyme |
| SMU_1904c | 1.8 | | Uncharacterized protein |
| SMU_1903c | 1.8 | | Uncharacterized protein |
| SMU_140 | 1.8 | | Putative glutathione reductase (EC 1.8.1.7) |
| SMU_152 | 1.8 | | Uncharacterized protein |
| SMU_153 | 1.8 | | Uncharacterized protein |
| SMU_148 | 1.7 | *adhE* | Aldehyde-alcohol dehydrogenase |
| SMU_1537 | 1.7 | *glgD* | Putative glycogen biosynthesis protein GlgD |
| SMU_139 | 1.7 | | Uncharacterized protein |
| SMU_172 | 1.7 | *mazE* | Uncharacterized protein |
| SMU_592c | 1.7 | | Putative transcriptional regulator |
| SMU_1495 | 1.7 | *lacB* | Galactose-6-phosphate isomerase subunit LacB (EC 5.3.1.26) |
| SMU_609 | 1.6 | | Putative 40K cell wall protein |
| SMU_1536 | 1.6 | *glgA* | Glycogen synthase (EC 2.4.1.21) (Starch [bacterial glycogen] synthase) |
| SMU_1538 | 1.6 | *glgC* | Glucose-1-phosphate adenylyltransferase (EC 2.7.7.27) (ADP-glucose pyrophosphorylase) (ADPGlc PPase) (ADP-glucose synthase) |
| SMU_807 | 1.6 | | Putative membrane protein |
| SMU_412c | 1.6 | | Putative Hit-like protein involved in cell-cycle regulation |
| SMU_1131c | 1.6 | | Uncharacterized protein |
| SMU_730 | 1.6 | | Uncharacterized protein |
| SMU_116 | 1.6 | *lacD2* | Tagatose 1,6-diphosphate aldolase 2 (EC 4.1.2.40) (D-tagatose-1,6-bisphosphate aldolase 2) (Tagatose-bisphosphate aldolase 2) |
| SMU_1862 | 1.6 | | Uncharacterized protein |
| SMU_1535 | 1.5 | *phsG* | Glycogen phosphorylase (EC 2.4.1.1) |
| SMU_1322 | 1.5 | *budC* | Putative acetoin dehydrogenase (EC 1.1.1.303) |
| SMU_1487 | 1.5 | | Uncharacterized protein |
| SMU_1490 | 1.5 | *lacG* | 6-phospho-beta-galactosidase (EC 3.2.1.85) (Beta-D-phosphogalactoside galactohydrolase) (PGALase) (P-beta-Gal) (PBG) |
| SMU_439 | 1.5 | | Putative transcriptional regulator |

| | | | |
|---|---|---|---|
| SMU_1492 | 1.5 | *lacF* | PTS system lactose-specific EIIA component (EC 2.7.1.207) (EIIA-Lac) (EIII-Lac) (Lactose-specific phosphotransferase enzyme IIA component) |
| SMU_1997 | 0.7 | *comX1* | Putative ComX1, transcriptional regulator of competence-specific genes |
| SMU_1805 | 0.66 | | Putative transcriptional regulator |
| SMU_2134 | 0.65 | | Putative transcriptional regulator |
| SMU_644 | 0.64 | | Putative competence protein/transcription factor |
| SMU_1006 | 0.6 | | Putative ABC transporter, ATP-binding protein |
| SMU_1007 | 0.59 | | Putative ABC transporter, permease protein |

**Table S13.** Altered gene expression common to all three stress conditions in wild-type strain (≥ 1.5-fold change, $P < 0.005$). Bolded gene IDs indicate altered gene expression observed in both wild-type and *lrgAB* mutant under all three conditions

a = aerobic, v = vancomycin, h = heat, ↑ = increased expression (WTx/WT), ↓ = decreased expression (ABx/AB)

| **Gene ID** | **Gene** | **Function** | **Direction of gene expression change** | | |
|---|---|---|---|---|---|
| | | | **a** | **v** | **h** |
| SMU_1001 | | Putative DNA processing Smf protein | ↓ | ↓ | ↓ |
| SMU_101 | | Putative sorbose PTS system, IIC component | ↑ | ↓ | ↑ |
| SMU_1013c | | Putative Mg2+/citrate transporter | ↑ | ↑ | ↑ |
| **SMU_114** | ***fruC*** | **Constitutive fructose permease (Putative PTS system, fructose-specific IIBC component)** | ↑ | ↑ | ↑ |
| **SMU_1400c** | | **Uncharacterized protein** | ↑ | ↑ | ↑ |
| **SMU_1419** | | **Putative transcriptional regulator** | ↓ | ↓ | ↓ |
| **SMU_1420** | | **Putative oxidoreductase (EC 1.6.5.2)** | ↓ | ↓ | ↓ |
| **SMU_1421** | ***pdhC*** | **Dihydrolipoamide acetyltransferase component of pyruvate dehydrogenase complex (EC 2.3.1.-)** | ↑ | ↑ | ↑ |
| **SMU_1422** | ***pdhB*** | **Putative pyruvate dehydrogenase E1 component beta subunit (EC 1.2.4.1)** | ↑ | ↑ | ↑ |
| **SMU_1423** | ***pdhA*** | **Putative pyruvate dehydrogenase, TPP-dependent E1 component alpha-subunit (EC 1.2.4.1)** | ↑ | ↑ | ↑ |
| **SMU_1584c** | | **Putative 67 kDa myosin-crossreactive streptococcal antigen-like protein** | ↓ | ↓ | ↓ |
| **SMU_1596** | ***ptcC*** | **Permease IIC component** | ↑ | ↑ | ↑ |
| SMU_1597c | | Uncharacterized protein | ↑ | ↑ | ↑ |
| SMU_1598 | *ptcA* | Putative PTS system, cellobiose-specific IIA component | ↑ | ↑ | ↑ |
| **SMU_1599** | ***celR*** | **Putative transcriptional regulator possible antiterminator** | ↑ | ↑ | ↑ |
| SMU_1914c | | Uncharacterized protein | ↑ | ↑ | ↓ |
| **SMU_1927** | | **Putative ABC transporter, ATP-binding protein** | ↓ | ↓ | ↓ |
| **SMU_2133c** | | **Putative membrane protein** | ↓ | ↓ | ↓ |
| SMU_29 | *purC* | Phosphoribosylaminoimidazole-succinocarboxamide synthase (EC 6.3.2.6) (SAICAR synthetase) | ↓ | ↓ | ↓ |
| SMU_498 | *comF* | Putative late competence protein | ↓ | ↓ | ↓ |
| SMU_574c | *lrgB* | Putative membrane protein | ↑ | ↑ | ↑ |
| SMU_625 | *comEA* | Putative competence protein | ↓ | ↓ | ↓ |
| SMU_626 | | Putative competence protein | ↓ | ↓ | ↓ |
| SMU_670 | *citB* | Aconitate hydratase A (ACN) (Aconitase) (2S,3R)-3-hydroxybutane-1,2,3-tricarboxylate dehydratase) (Iron-responsive protein-like) (IRP-like) (Probable 2-methyl-cis-aconitate hydratase) (RNA-binding protein) | ↓ | ↑ | ↓ |
| SMU_671 | *citZ* | Citrate synthase (EC 2.3.3.16) | ↓ | ↑ | ↓ |
| SMU_672 | *icd* | Isocitrate dehydrogenase [NADP] (IDH) (EC 1.1.1.42) (IDP) (NADP(+)-specific ICDH) (Oxalosuccinate decarboxylase) | ↓ | ↑ | ↓ |

**Table S14.** Altered gene expression common to all three stress conditions in *lrgAB* mutant (≥ 1.5-fold change, $P < 0.005$). Bolded gene IDs indicate altered gene expression observed in both wild-type and lrgAB mutant under all three conditions

a = aerobic, v = vancomycin, h = heat, ↑ = increased expression (ABx/AB), ↓ = decreased expression (ABx/AB)

| Gene ID | Gene | Function | Direction of gene expression change | | |
|---|---|---|---|---|---|
| | | | a | v | h |
| **SMU_114** | ***fruC*** | **Constitutive fructose permease (Putative PTS system, fructose-specific IIBC component)** | ↑ | ↑ | ↑ |
| SMU_116 | *lacD2* | Tagatose 1,6-diphosphate aldolase 2 (EC 4.1.2.40) (D-tagatose-1,6-bisphosphate aldolase 2) (Tagatose-bisphosphate aldolase 2) | ↑ | ↑ | ↑ |
| SMU_140 | | Putative glutathione reductase (EC 1.8.1.7) | ↑ | ↑ | ↑ |
| **SMU_1400c** | | **Uncharacterized protein** | ↑ | ↑ | ↑ |
| **SMU_1419** | | **Putative transcriptional regulator** | ↓ | ↓ | ↓ |
| **SMU_1420** | | **Putative oxidoreductase (EC 1.6.5.2)** | ↓ | ↓ | ↓ |
| **SMU_1421** | ***pdhC*** | **Dihydrolipoamide acetyltransferase component of pyruvate dehydrogenase complex (EC 2.3.1.-)** | ↑ | ↑ | ↑ |
| **SMU_1422** | ***pdhB*** | **Putative pyruvate dehydrogenase E1 component beta subunit (EC 1.2.4.1)** | ↑ | ↑ | ↑ |
| **SMU_1423** | ***pdhA*** | **Putative pyruvate dehydrogenase, TPP-dependent E1 component alpha-subunit (EC 1.2.4.1)** | ↑ | ↑ | ↑ |
| SMU_148 | *adhE* | Aldehyde-alcohol dehydrogenase | ↑ | ↑ | ↑ |
| SMU_1487 | | Uncharacterized protein | ↑ | ↑ | ↑ |
| SMU_1490 | *lacG* | 6-phospho-beta-galactosidase (EC 3.2.1.85) (Beta-D-phosphogalactoside galactohydrolase) (PGALase) (P-beta-Gal) (PBG) | ↑ | ↑ | ↑ |
| SMU_1493 | *lacD* | Tagatose 1,6-diphosphate aldolase 1 (EC 4.1.2.40) (D-tagatose-1,6-bisphosphate aldolase 1) (Tagatose-bisphosphate aldolase 1) | ↑ | ↑ | ↑ |
| SMU_150 | | Uncharacterized protein | ↑ | ↑ | ↑ |
| SMU_151 | | Uncharacterized protein | ↑ | ↑ | ↑ |
| SMU_1535 | *phsG* | Glycogen phosphorylase (EC 2.4.1.1) | ↑ | ↑ | ↑ |
| SMU_1536 | *glgA* | Glycogen synthase (EC 2.4.1.21) (Starch [bacterial glycogen] synthase) | ↑ | ↑ | ↑ |
| SMU_1537 | *glgD* | Putative glycogen biosynthesis protein GlgD | ↑ | ↑ | ↑ |
| SMU_1538 | *glgC* | Glucose-1-phosphate adenylyltransferase (EC 2.7.7.27) (ADP-glucose pyrophosphorylase) (ADPGlc PPase) (ADP-glucose synthase) | ↑ | ↑ | ↑ |
| **SMU_1584c** | | **Putative 67 kDa myosin-crossreactive streptococcal antigen-like protein** | ↑ | ↑ | ↓ |
| **SMU_1596** | ***ptcC*** | **Permease IIC component** | ↑ | ↑ | ↑ |
| **SMU_1599** | ***celR*** | **Putative transcriptional regulator possible antiterminator** | ↑ | ↑ | ↑ |
| SMU_1601 | *bgl* | Putative phospho-beta-glucosidase (EC 3.2.1.86) | ↑ | ↑ | ↑ |
| SMU_1602 | | Putative NAD(P)H-flavin oxidoreductase | ↑ | ↑ | ↑ |
| SMU_1603 | *lguL* | Putative lactoylglutathione lyase (EC 4.4.1.5) | ↑ | ↑ | ↑ |
| SMU_1862 | | Uncharacterized protein | ↑ | ↑ | ↑ |
| **SMU_1927** | | **Putative ABC transporter, ATP-binding protein** | ↓ | ↓ | ↓ |
| SMU_1997 | *comX1* | Putative ComX1, transcriptional regulator of competence-specific genes | ↓ | ↓ | ↓ |
| **SMU_2133c** | | **Putative membrane protein** | ↓ | ↓ | ↓ |
| SMU_609 | | Putative 40K cell wall protein | ↑ | ↑ | ↑ |
| SMU_652c | | Putative ABC transporter, ATP-binding protein possible nitrate transport system | ↑ | ↑ | ↑ |
| SMU_932 | | Uncharacterized protein | ↓ | ↑ | ↓ |

| | | | | | |
|---|---|---|---|---|---|
| SMU_933 | | Putative amino acid ABC transporter, periplasmic amino acid-binding protein | ↓ | ↑ | ↓ |
| SMU_934 | | Putative amino acid ABC transporter, permease protein | ↓ | ↑ | ↓ |
| SMU_935 | | Putative amino acid ABC transporter, permease protein | ↓ | ↑ | ↓ |
| SMU_936 | | Putative amino acid ABC transporter, ATP-binding protein | ↓ | ↑ | ↓ |
| SMU_961 | *ahpD* | Alkyl hydroperoxide reductase AhpD (EC 1.11.1.15) | ↓ | ↑ | ↓ |
| SMU_962 | | Putative dehydrogenase | ↓ | ↑ | ↓ |
